# Effect of Adjunct Metformin Treatment in Patients with Type-1 Diabetes and Persistent Inadequate Glycaemic Control. A Randomized Study

**DOI:** 10.1371/journal.pone.0003363

**Published:** 2008-10-09

**Authors:** Søren Søgaard Lund, Lise Tarnow, Anne Sofie Astrup, Peter Hovind, Peter Karl Jacobsen, Amra Ciric Alibegovic, Ida Parving, Lotte Pietraszek, Merete Frandsen, Peter Rossing, Hans-Henrik Parving, Allan Arthur Vaag

**Affiliations:** 1 Steno Diabetes Center, Gentofte, Denmark; 2 Rigshospitalet, Department of Medical Endocrinology, University of Copenhagen, Copenhagen, Denmark; 3 Faculty of Health Sciences, University of Aarhus, Aarhus, Denmark; 4 Department of Endocrinology, University of Lund, Malmö, Sweden; London School of Hygiene and Tropical Medicine, Peru

## Abstract

**Background:**

Despite intensive insulin treatment, many patients with type-1 diabetes (T1DM) have longstanding inadequate glycaemic control. Metformin is an oral hypoglycaemic agent that improves insulin action in patients with type-2 diabetes. We investigated the effect of a one-year treatment with metformin versus placebo in patients with T1DM and persistent poor glycaemic control.

**Methodology/Principal Findings:**

One hundred patients with T1DM, preserved hypoglycaemic awareness and HaemoglobinA_1c_ (HbA_1c_) ≥8.5% during the year before enrolment entered a one-month run-in on placebo treatment. Thereafter, patients were randomized (baseline) to treatment with either metformin (1 g twice daily) or placebo for 12 months (double-masked). Patients continued ongoing insulin therapy and their usual outpatient clinical care. The primary outcome measure was change in HbA_1c_ after one year of treatment. At enrolment, mean (standard deviation) HbA_1c_ was 9.48% (0.99) for the metformin group (n = 49) and 9.60% (0.86) for the placebo group (n = 51). Mean (95% confidence interval) baseline-adjusted differences after 12 months with metformin (n = 48) versus placebo (n = 50) were: HbA_1c_, 0.13% (−0.19; 0.44), p = 0.422; Total daily insulin dose, −5.7 U/day (−8.6; −2.9), p<0.001; body weight, −1.74 kg (−3.32; −0.17), p = 0.030. Minor and overall major hypoglycaemia was not significantly different between treatments. Treatments were well tolerated.

**Conclusions/Significance:**

In patients with poorly controlled T1DM, adjunct metformin therapy did not provide any improvement of glycaemic control after one year. Nevertheless, adjunct metformin treatment was associated with sustained reductions of insulin dose and body weight. Further investigations into the potential cardiovascular-protective effects of metformin therapy in patients with T1DM are warranted.

**Trial Registration:**

ClinicalTrials.gov NCT00118937

## Introduction

Despite intensive insulin treatment and patient care, many patients with type-1 diabetes have longstanding poor metabolic control and their glycaemic levels remain substantially higher than the recommended target of HaemoglobinA_1c_ (HbA_1c_) less than 7.0% [1], [2]. As recently reviewed by deVries et al., the phenomenon of persistent poor glycaemic control seems to have multiple and complex causes and, so far, no simple, single solution to this problem has emerged [2]. The Diabetes Control and Complication Trial demonstrated the beneficial effect of improving glycaemic control on the long-term risk of late-diabetic complications [3], [4]. Decreased muscle glucose uptake in response to insulin (i.e., decreased insulin action, otherwise known as “insulin resistance”) has been demonstrated in patients with type-1 diabetes [5] and this might contribute to the development and maintenance of their poor glycaemic control.

Metformin is an oral anti-hyperglycaemic agent that has been extensively used in the treatment of patients with type-2 diabetes. Enhanced insulin action and decreased hepatic glucose output have been suggested as the primary modes of action of metformin [6]. For the same degree of blood glucose reduction, metformin treatment is associated with a lower risk of hypoglycaemia than with insulin secretagogues and insulin treatment in patients with type-2 diabetes [7], [8]. Metformin also has a beneficial effect on the risk of macrovascular complications in obese patients with type-2 diabetes [8].

Previous studies have demonstrated an insulin-sparing effect of metformin treatment as an adjunct therapy to ongoing insulin treatment in patients with type-1 diabetes. In contrast, the results concerning glycaemic control and other metabolism-related variables (e.g., body weight) are contradictory. Marked differences between studies in baseline levels of glycaemic control (for example, HbA_1c_ ranged from 7.6% to 10.9%), relatively small sample sizes and short duration of interventions (six months or less) could contribute to such discrepancies [9]–[24]. Hence, the effect of metformin treatment on glycaemic control and other cardiovascular risk factors in patients with type-1 diabetes remains a matter of controversy. We hypothesized that metformin treatment might reduce glycaemia as well as other non-glycaemic cardiovascular risk markers in patients with type-1 diabetes and persistent poor glycaemic control. Here, we report the results of a one-year trial of metformin versus placebo as an adjunct therapy in 100 patients with type-1 diabetes and persistent poor glycaemic control.

## Methods

The protocol for this trial and supporting CONSORT checklist are available as supporting information; see Checklist S1, Protocol S1, Protocol S2, Amendment S1.

### Study design

The study was an investigator-initiated, single-centre, randomized, double-masked, parallel trial of metformin versus placebo treatments.

### Participants

Inclusion, exclusion and withdrawal criteria are presented in Table 1. The data were collected at Steno Diabetes Center, Gentofte, Denmark.

**Table 1:** Inclusion, exclusion and withdrawal criteria

*Inclusion criteria:*
• Type-1 diabetes mellitus according to the World Health Organization 1999 definition^a^, including age at onset of diabetes ≤35 years and a non-fasting^b^ serum C-peptide ≤300 pmol/l.
• Diabetes duration≥5.0 years.
• Age at enrolment≥18.0 years.
• Mean HbA_1c_≥8.5% including all available measurements during one year before enrolment and HbA_1c_≥8.5% at enrolment.
***Exclusion and withdrawal criteria:***
• HbA_1c_<8.0% at baseline (0-month study visit).
• Hypoglycaemic unawareness.
• Clinical signs of heart failure.
• Plasma creatinine above the normal upper limit.
• Plasma AST elevated more than three-fold above the normal upper limit.
• Factor II VII X decreased<0.7.
• Serious co-morbidities.
• Pregnancy.
• History of drug or alcohol abuse.
• Unexpected serious adverse events with potential relation to the study drug.

a See reference [33].

b At enrolment, the level of C-peptide was determined in non-fasting samples. In the case of non-fasting C-peptide levels≥300 pmol/l, a fasting sample and/or a glucagon-stimulated C-peptide measure was obtained to evaluate beta cell function. According to local guidelines, suggested cut-off levels for C-peptide indicative of type-1 diabetes were: Fasting<300 pmol/l; after 1 mg i.v. glucagon: ≤600 pmol/l.

Abbreviations:

HbA_1c_: HaemeglobinA_1c_

AST: Aspartate aminotransferase

### Ethics

At the screening visit (−1 month) patients gave written informed consent. The study was conducted in accordance with the Declaration of Helsinki and was approved by the Ethics Committee of Copenhagen County, Denmark.

### Interventions

Patients continued ongoing insulin therapy as well as their usual outpatient clinical care throughout the study period (i.e., outpatient clinic visits were planned independently of patients' participation in the study). Adjustments in life-style, home-monitored blood glucose measurements, insulin dose and concomitant medications were made at the discretion of the patients and the clinicians in the outpatient clinic. After the screening visit, patients entered a one-month run-in period, during which they began a single-masked treatment with one placebo tablet per day. Patients with a level of HbA_1c_≥8.0% at the end of the run-in period were randomized to continue ongoing insulin therapy plus either metformin or placebo tablets for 12 months. The dose of metformin or placebo was gradually increased by forced titration once weekly (the starting dose of metformin was 500 mg once daily) reaching the maximum dose (1 g twice daily, i.e., 2 g total daily dose) after three weeks. Doses were reduced if adverse events occurred that could possibly have been associated with the study medication. Once free of adverse events, the drug dose was increased again and, if adverse events recurred, the lower dose was resumed. Otherwise, through four regular scheduled telephone consultations (at one and three months post-randomization and hereafter approximately every third to fourth month) the study nurse ensured adequate study medication dose titration. Active and placebo tablets were identical in appearance, taste and smell. The near-maximum dose of metformin was chosen since previous dose-response studies showed only slight additional lowering of blood glucose and a tendency for more side effects with higher doses of metformin [25]. Patients were advised to take tablets just before or during their morning and evening meals.

### Objectives

The objective of the trial was to assess the efficacy and safety of adjunct metformin treatment during one year in adult patients with type-1 diabetes and persistent poor glycaemic control.

### Outcomes

#### Primary outcome

The level of HbA_1c_ after 12 months intervention.

#### Secondary outcomes

Pre-specified secondary outcomes were additional variables related to glycaemic control (fasting plasma glucose, insulin dose, body-weight, waist- and hip-circumferences and adverse events including hypoglycaemia). Safety variables included blood concentrations of haemoglobin, creatinine, sodium, potassium, bicarbonate, cobalamin, folate, alkaline phosphatase, aspartate aminotransferase as well as platelets and white blood cell counts. Compliance with the study medication. Endpoints were assessed at enrolment (screening visit: −1-month visit), in the week before randomization (baseline, 0-month visit) and on the last treatment day (end of treatment: 12-month visit). Patients were asked to report immediately important medical occurrences. Otherwise, through the four telephone consultations (see above), the study nurse collected intermediate information about insulin doses and adverse events, including hypoglycaemia. Information about type and dose of concomitant medications was collected at each of the three study visits (screening, baseline and end of treatment).

#### Ancillary outcomes

The change in HbA_1c_ during the run-in period. Intermediate HbA_1c_ measurements collected from visits in the outpatient clinic between baseline and end of treatment visits. Insulin doses at the three months telephone consultations. The number of outpatient clinic visits during follow-up. Unconsciousness during hypoglycaemic events. Self-reported blood/plasma glucose levels during hypoglycaemic events.

#### Sample size

The study aimed to show the superiority of metformin over placebo to influence the level of HbA_1c_ and was designed with a statistical power of 80% to detect a 0.60% absolute HbA_1c_ difference. This value was equal to the difference reported between the conventional and metformin-treated groups in the UK Prospective Diabetes Study [8]. There was an estimated standard deviation (SD) of 1.0% for a two-sided 5% significance level. Accordingly, 100 enrolled subjects were needed if up to ten drop-outs were to occur.

#### Randomization–Sequence generation

Randomization was performed using a pre-established computer-generated sequence in blocks of three and four, with the block size masked during the trial. Patients were stratified into four groups according to the baseline level of HbA_1c_ (≤ or >9.5%) and BMI (< or ≥25 kg/m^2^).

#### Randomization–Allocation concealment

Double-masked treatments were allocated using numbered drug containers. Concealed treatment allocation was by central randomization (by email correspondence). KLIFO A/S (Copenhagen, Denmark) secured masking during the trial. A sealed copy of the randomization sequence was available at the investigation site in case of need for emergency unblinding.

#### Randomization–Implementation

KLIFO A/S established the sequence. The trial clinicians enrolled patients. A person unconnected with the study assigned participants to their groups.

#### Blinding

Treatment allocation was masked for participants, clinicians and those evaluating outcomes until after database lock. After close-out the code was two-step encrypted (first step: treatment A or B; second step: A = metformin and B = placebo). The success of the masking was assessed by patients and clinicians estimating allocated treatments at end of treatment (without knowledge of the treatment actually allocated or the other's answer).

### Blood sampling

At baseline and 12 month study visits, blood samples were drawn in the fasting state. At enrolment, blood was collected as random, non-fasting samples.

### Biochemical and other analyses

HbA_1c_ was measured by the ion-exchange HPLC-method traceable to The Diabetes Control and Complication Trial standard (Tosoh Bioscience, Minato, Japan; normal limits: 4.1–6.4%; intra/inter assay SD of 0.04/0.10, respectively). HbA_1c_ was measured twice on each study visit and the mean of the measurements of the two separately drawn blood samples was used in all statistical analyses (mean [SD] absolute intra-subject difference between HbA_1c_ measurements, in percentage points, baseline: 0.11% [0.11]; end of treatment: 0.14% [0.12]; corresponding to a mean [SD] intra-subject difference relative to the mean of the two measurements, baseline: 0.6% [0.6%]; end of treatment: 0.7% [0.6%]). Serum C-peptide was measured with time-resolved fluoroimmunoassay (AutoDELFIA, PerkinElmer, MA, USA; lower limit of detection: 10 pmol/l). Office arterial blood pressure was assessed with a UA-779 automatic digital device (A&D Instruments LTD., Abingdon, Oxon, UK), taking one measurement on each arm after a minimum of 5 min at rest, seated upright. The mean of the two measurements was analyzed. Safety variables were measured using routine procedures at the Steno Diabetes Center. Body weight and height as well as waist and hip circumference were measured with the patient standing upright wearing only underwear. Body mass index (BMI) was calculated as body weight/height^2^ (kg/m^2^). At study visits, plasma glucose was measured with the glucose-oxidase biosensor method (Medisense Precision Xtra™ hand-device, ABBOTT Laboritories, Bedford, MA 017301, USA). At the periodical recordings (i.e., at telephone contacts and at scheduled study visits) patients were asked to estimate the average level of home-monitored blood/plasma glucose measurements during hypoglycaemic events. Otherwise, home-monitored blood/plasma glucose measurements were not captured during the study. At enrolment, patients were asked to choose from one or more of five categories of reasons for having unsatisfactory glycaemic control: fear of hypoglycaemia; non-compliance with diet; insufficient physical activity; technical difficulties, for example, with insulin injections; other reasons.

### Adverse events

Information about adverse events was collected with a standardised questionnaire. A serious adverse event (SAE) was defined as one fulfilling any of the following criteria: any untoward medical occurrence that at any dose: resulted in death; was life-threatening, required inpatient hospitalisation or prolongation of existing hospitalisation; resulted in persistent or significant disability/incapacity or was a congenital anomaly or birth defect. Minor episodes of hypoglycaemia were considered to be those in which the hypoglycaemic symptoms were treated by the patient; major hypoglycaemic episodes were considered to be those in which the condition induced loss of consciousness or when third-party intervention was required to obtain treatment. Events of unconsciousness during major hypoglycaemia were captured using a separate category for this in the case-report form.

### Compliance

Compliance and drug exposure during the study were assessed by tablet counting. Compliance with the tablet consumption schedule was calculated as the actual study drug consumption as a percentage of the expected consumption according to the currently prescribed study drug dose. Study drug exposure was calculated as the actual consumption averaged over the total number of days between the first and last study visits in the treatment periods. The number of outpatient clinic visits was estimated by the number of HbA_1c_ measurements between (i.e., excluding) study visits.

### Statistical methods

#### Subject characteristics

At enrolment, normally distributed variables were compared using unpaired t-tests, whereas non-normally distributed variables were analyzed with the Mann-Whitney test. Categorical data were analyzed using Fisher's exact test or, in case of more than two categories, by Pearsons chi-square test.

#### Primary outcome, primary analysis

Differences in treatment effects between the randomized interventions were evaluated by comparison between end of treatment and baseline levels (i.e., “change from baseline”). An analysis of covariance (ANCOVA) model was developed with treatment type (metformin or placebo) as the fixed effect and baseline level as the covariate.

#### Primary outcome, secondary analyses

Further pre-specified analyses of the primary outcome, HbA_1c_, included adjustment for the current (i.e., baseline and on-treatment) insulin dose as well as interaction analyses of subgroups. The following subgroups of patients were analyzed: (A) Substantial changes in the insulin regimens during the trial (i.e., changes of insulin preparations, for example, switching from human insulin to insulin analogues, or vice versa [metformin: n = 8, placebo: n = 8], changes in the number and/or the site of daily insulin injections of one or more insulin preparations [metformin: n = 3; placebo: n = 6], or changes in insulin preparations as well the number/site of injections [metformin: n = 1; placebo: n = 3]). (B) Patients according to dichotomized baseline levels of BMI (< or ≥25 kg/m^2^), median baseline levels of HbA_1c_ (≤ or >9.15%) or of total daily insulin dose (< or ≥0.72 U/kg). The dichotomization of BMI, HbA_1c_ and insulin dose was done since these patient characteristics have been proposed as markers of insulin resistance–a suggested target of metformin treatment. Hence, any interaction of treatment by subgroups of patients according to the dichotomized baseline levels of these variables could potentially discriminate responders to non-responders of metformin therapy.

#### Secondary outcomes, primary analysis

Continuous variables were analyzed using the baseline adjusted ANCOVA model as for the primary outcome. Compliance as well as the number of SAE's were analyzed with the Mann-Whitney test. Categorical data were analyzed using Fisher's exact test.

#### Ancillary analyses

The HbA_1c_ measurements were investigated during the run-in period with adjustment for HbA_1c_ at enrolment. Due to the observed differences in lipid-lowering therapies at enrolment/baseline, that is, statin and fish-oil therapies, additional adjustment for these differences were performed for the primary outcome, HbA_1c_. All follow-up (i.e., intermediate from outpatient clinic visits as well as end of treatment) HbA_1c_ measurements at least one month post-randomization (a total of 354 HbA_1c_ measurements) were evaluated in a repeated measures analysis (mixed model with fixed effects: baseline HbA_1c_, treatment, time and time by treatment interaction; random effects: subject and subject by time interaction; covariance structure: variance components) [26]. Subsequently, the first and the last available of these follow-up HbA_1c_ data as well as insulin dose after three months, were analyzed as for the primary outcome.

In addition to hypoglycaemia being evaluated as a categorical variable (i.e., the number of patients reporting events), the number of events was evaluated as a continuous variable (i.e., the number of hypoglycaemic events was considered equidistant). Absolute numbers of hypoglycaemic events during follow-up as well as changes from enrolment or the run-period were evaluated. The number of hypoglycaemic events and outpatient clinic visits during follow-up (i.e., the number of HbA_1c_ measurements) as well as the self-reported blood/plasma glucose levels during hypoglycaemic events were analyzed with the Mann-Whitney test (for comparisons between subjects) or the Wilcoxon signed rank test (for comparisons within subjects). Categorical data were analyzed as for the secondary outcomes.

Data for primary, secondary and ancillary outcomes are summarised either as means (SD) for normally distributed variables or as medians and ranges for non-normally distributed variables. Treatment effects estimated by the model are presented as means and associated 95% confidence intervals. In the ANCOVA model, variables showed normally distributed residuals. No corrections for multiple testing were performed.

Statistical analyses were done with SPSS, version 14.0 (Chicago, Illinois, USA).

#### Protocol deviations (with relation to the follow-up of patients)

In addition to the ancillary analyses as outlined, the following patient related protocol deviations were noted (see supplementary files Protocol S1 and S2 as well as Amendment S1 for details). Three screen-failure patients were included in the analyses. One patient being diagnosed with diabetes after the age of 35 years. The patient fulfilled all other inclusion criteria (the patient was 37 years old at diagnosis of diabetes and had a level of non-fasting C-peptide <10 pmol/l). At enrolment, the second screen-failure patient presented with HbA_1c_ of 8.30% (both measurements) and, due to diabetic nephropathy, elevated plasma creatinine of 117 pmol/l (reference range: 62–100 μmol/l). In this patient, the creatinine level normalized during the run-in period and all other inclusion criteria were fulfilled (mean HbA_1c_ was 9.7% during the year before enrolment and, at randomization, the patient presented with HbA_1c_ measurements of 8.7% and 8.6%, respectively). The third screen-failure patient presented with increased levels of creatinine due to diabetic nephropathy (at enrolment plasma creatinine was 120 μmol/l; at randomization plasma creatinine was 105 μmol/l; reference range: 62–100 μmol/l). At enrolment, a total of five more patients presented with levels of either creatinine (n = 1) or factor II VII X (n = 4) outside the reference ranges, but in whom these variables normalized during the run-in period. Due to adverse gastrointestinal events during the run-in period (i.e., during single-masked placebo treatment), one patient (in the metformin group) was unmasked to that treatment (i.e., run-in placebo). Otherwise no preterm unmasking occurred. One patient in the placebo group, who experienced a SAE with potential relation to the study drug (i.e., ketoacidosis precipitated by gastroenteritis–see below) was not withdrawn from the study (one more patient, in the placebo group, who experienced a SAE due to ketoacidosis–see below–dropped out while hospitalized herefore).

## Results

### Subjects

**Figure 1:**
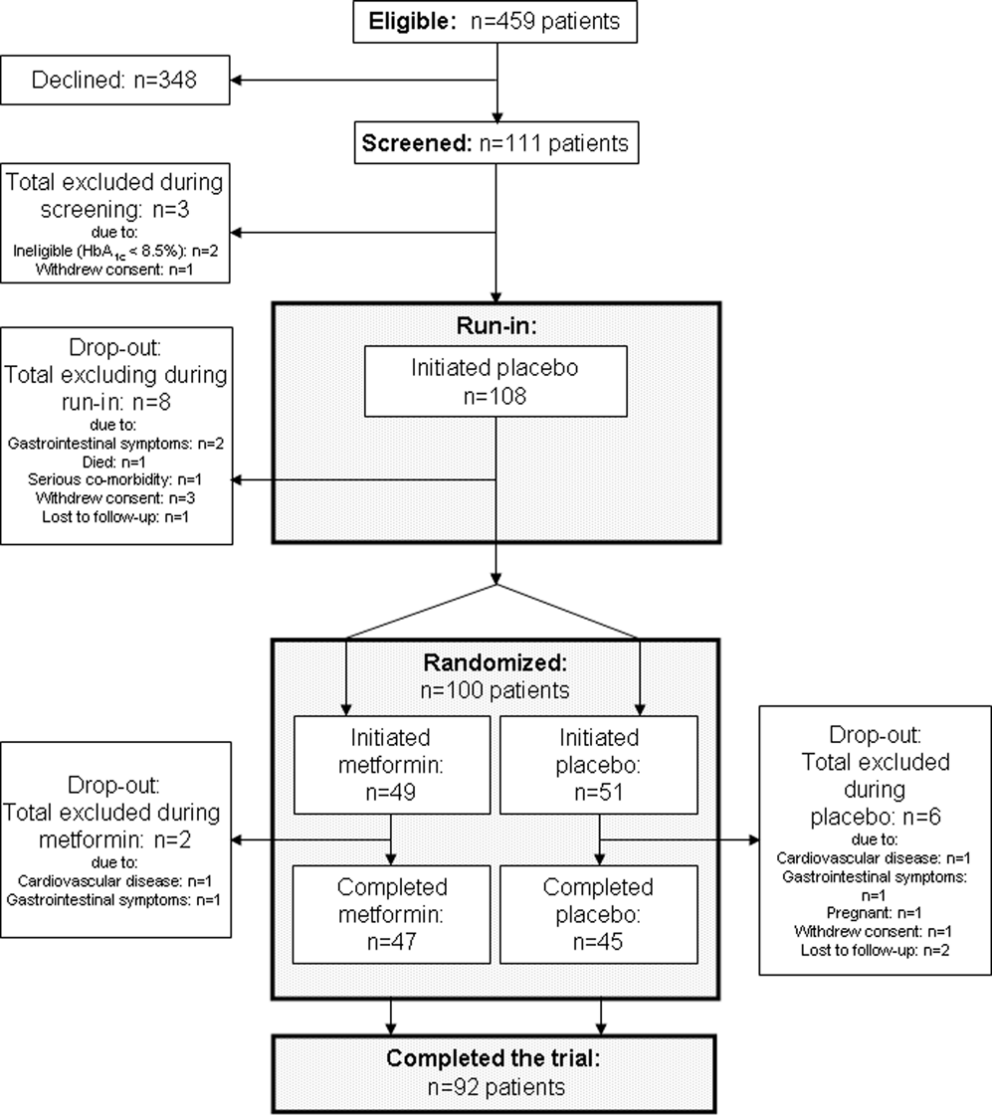
Patient flow scheme.

All potentially eligible patients with type-1 diabetes at the Steno Diabetes Center (n = 459) were invited to participate. 111 patients accepted (24%). Eleven patients were excluded before randomization. Thus, a total of 100 patients were randomized for the study medication, of which 49 began treatment with metformin and 51 received placebo. Two patients [4.1%] were excluded during metformin treatment and six patients [11.8%] dropped out from the placebo group. Thus, 47 and 45 patients [95.9% and 88.2%] completed the 12-month treatment period with metformin or placebo, respectively (Figure 1).

#### Recruitment

Patients were enrolled from August 2004 through June 2005 and follow-up ended August 2006.

### Subject characteristics

Among those invited patients who declined (n = 348), approximately half were male compared to approximately two-thirds of those patients who accepted and performed the screening visit (n = 111) (p = 0.037). Otherwise, at time of approach, age, known duration of diabetes and the level of HbA_1c_ were not significantly different between the two groups (data not shown).

The clinical characteristics of study subjects at enrolment (n = 100) are summarized in Table 2. All patients were Caucasians, and about two-thirds of them were male. Their mean age was approximately 45 years with a median known duration of diabetes of 25 to 30 years and a mean BMI of approximately 26 kg/m^2^. Most patients received four daily insulin injections, thereby receiving a daily dose of approximately 60 units. For the year before and at enrolment, HbA_1c_ was 9.5 to 9.6%. Non-compliance with diet was the most frequently reported cause of persistent unsatisfactory glycaemic control. Very few patients reported major hypoglycaemic events within the preceding year. Except for greater numbers of patients who received ongoing statin or fish-oil therapies in the metformin group (p = 0.039 and p = 0.007, respectively), the randomization procedure produced treatment groups that were well matched for metabolic and clinical characteristics (Table 2). Six patients or fewer within each non-study medication category, as listed in Table 2, started and/or stopped this medication during the trial.

**Table 2:** Subject characteristics at enrolment (−1 month) for 100 patients with type-1 diabetes.

*Subject characteristics*	Allocated metformin (n = 49)	Allocated placebo (n = 51)	p-value
***Gender*** (men/women)	**33** [67]**/16** [33]	**31** [61]**/20** [39]	0.537
***Age*** (years)	**46.1** (11.6)	**44.9** (10.8)	0.592
***Known duration of diabetes*** * (years)*	**30** (5; 51)	**26** (6; 56)	0.775
***Body weight*** (kg)	**80.5** (12.5)	**79.0** (15.3)	0.600
***Height*** (m)	**1.75** (0.10)	**1.75** (0.09)	0.735
***Body mass index*** (kg/m^2^)	**26.2** (3.4)	**25.8** (4.3)	0.642
***HaemoglobinA_1c_*** (%)	**9.48** (0.99)	**9.60** (0.86)	0.498
***HaemoglobinA_1c_*** * during the year before enrolment* (%)	**9.62** (0.83)	**9.63** (0.67)	0.947
***Non-fasting serum C-peptide*** (pmol/l)	**<10** (<10; 188)	**<10** (<10; 226)	0.712
***Office*** ** ***systolic blood pressure*** (mmHg)	**137.4** (18.2)	**140.1** (17.4)	0.449
***Office diastolic blood pressure*** (mmHg)	**78.9** (11.4)	**81.1** (9.9)	0.312
***Heart rate*** (beats/minute)	**76.7** (13.6)	**75.4** (12.1)	0.605
***Smoking*** (no/former/yes, daily or occasional)	**19** [39]/**11** [22]/**19** [39]	**26** [51]**/7** [14] **/18** [35]	0.374
***Alcohol*** (drinks per day: none/1-3/>3)^a^	**13** [50]/**13** [50]/**0** [0]	**14** [52]/**13** [48]/**0** [0]	− b
*Late diabetic complications:*			
***Retinopathy*** (none/simplex/proliferative)	**4** [8] **/25** [51]**/20** [41]	**6** [12] **/24** [47]**/21** [41]	0.817
***Macroangiopathy*** (no/yes)^c^	**39** [80]**/10** [20]	**45** [88]**/6** [12]	0.283
***Nephropathy*** (normo-/micro-/macroalbuminuria)^d^	**31** [63]**/11** [22] **/7** [14]	**29** [57]**/16** [31] **/6** [12]	0.598
***Neuropathy*** (no/yes)^e^	**25** [51]**/24** [49]	**28** [55]**/23** [45]	0.841
*Pre-study insulin therapy and hypoglycaemia:*			
***Total daily insulin dose*** (Units)/(Units/kg)	**59.8/0.74** (23.4/0.26)	**59.1/0.75** (19.2/0.22)	0.866
***No. of daily insulin injections*** (<4 daily/4 daily)^f^	**5** [10] **/44** [90]	**8** [16] **/43** [84]	0.555
***Minor hypoglycaemia*** * (No. of events during the last month)*	**5** (0; 20)	**4** (0; 20)	0.505
***Major hypoglycaemia*** * (No. of events during the last year)*	**0** (0; 3)	**0** (0; 10)	0.756
*Reported reasons for inadequate glycaemic control:*			
***To avoid hypoglycaemia***	**16** [33]	**15** [29]	0.830
***Non-compliance with diet***	**23** [47]	**24** [47]	− b
***Insufficient physical activity***	**15** [31]	**19** [37]	0.531
***Difficulties with insulin injections***	**5** [10]	**5** [10]	− b
***Other reasons***	**19** [39]	**23** [45]	0.549
*Ongoing non-study medication:*			
***Total on antihypertensive medication***	**30** [61]	**28** [55]	0.549
*** ACE inhibitors or angiotensin II receptor blockers***	**28** [57]	**24** [47]	0.326
*** Aldosterone antagonists***	**0** [0]	**0** [0]	−
*** Thiazide or loop diuretics*** g	**17** [35]	**15** [29]	0.669
*** Beta blockers***	**4** [8]	**3** [6]	0.712
*** Calcium channel blockers***	**6** [12]	**9** [18]	0.578
*** Other antihypertensive medication***	**0** [0]	**0** [0]	−
***Total on lipid-lowering medication***	**26** [53]	**15** [29]	**0.025**
*** Statins*** g	**23** [47]	**14** [27]	0.062
*** Fibrates***	**0** [0]	**0** [0]	−
*** Fish oil***	**9** [18]	**1** [2]	**0.007**
*** Other lipid-lowering medication***	**0** [0]	**0** [0]	−
***Other:***			
*** Aspirin***	**18** [37]	**15** [29]	0.525
*** Non-steroid anti-inflammatory drugs***	**3** [6]	**3** [6]	−
*** Dietary supplementations*** g, h	**4** [8]	**1** [2]	0.200
*** Cobalamin supplementations***	**0** [0]	**1** [2]	− b
*** Folic acid supplementations***	**0** [0]	**0** [0]	−
*** Potassium supplementations*** g	**14** [29]	**12** [24]	0.651
*** Thyroxine***	**3** [6]	**6** [12]	0.488

Data represent number of patients [%], mean (standard deviation) or median (range).

a Information about daily alcohol intake was missing in 23 and 24 patients allocated metformin and placebo, respectively. The percentage of patients refers to those for whom data were available.

b Not compared statistically due to equal proportions of patients or too few patients in treatment groups.

c Previous ischaemic heart disease, stroke, transient ischaemic attack or peripheral arterial disease.

d Normo-, micro- and macro-albuminuria: 24-hour urinary albumin-excretion≤29 mg, 30–299 mg and ≥300 mg, respectively, in two out of three consecutive samples. One patient, in the metformin group, had a kidney transplant five years prior to enrolment due to polycystic kidney disease. The patient presented with normal levels of creatinine and microalbuminura. Due to having prior kidney disease, the patient is included in the macroalbuminuria group.

e Symptomatic peripheral or autonomic neuropathy or clinical signs thereof.

f One patient, in the metformin group, received continuous subcutaneous insulin-infusions. The patient is included in the group with four daily injections.

g During the run-in period, in the metformin group, one patient changed from thiazid to loop diuretics, one patient initiated statin treatment and two patients initiated dietary (n = 1) or potassium (n = 1) supplementations, respectively, whereas, in the placebo group, one patient was temporarily treated with non-steroid anti-inflammatory drugs. Thus, at baseline (0 months), in the metformin and placebo groups, respectively, a total of 24 (49%) and 14 (27%) patients received ongoing statin treatment (**p = 0.039** between treatments); five (10%) and one (2%) patients received dietary supplementations (p = 0.108 between treatments) and 15 (31%) and 12 (24%) patients received potassium supplementations (p = 0.502 between treatments). In contrast, for the whole group of thiazid or loop diuretics as well as for non-steroid anti-inflammatory drugs, despite changes herein during the run-in period, a similar number of patients received such treatments at baseline compared to enrolment. Otherwise, patients did not start and/or stop other non-study medications as listed during the run-in period.

h Over-the-counter vitamin pills with unspecified cobalamin and folic acid content.

Abbreviation: ACE: Angiotensin converting enzyme.

Among excluded patients, mean HbA_1c_ at screening was 8.93% in the metformin group (n = 2) and 10.04% in the placebo group (n = 6).

#### Numbers analyzed, primary outcome

As pre-specified, the primary outcome was analyzed as intention-to-treat: all randomized patients exposed to the study medication with HbA_1c_ data available after a minimum of three months treatment (metformin: n = 48; placebo: n = 50). The intention-to-treat analysis included last observation carried forward (LOCF) for missing values at end of treatment.

#### Numbers analyzed, secondary outcomes

The secondary outcomes were analyzed similarly as for the primary outcome, that is, including LOCF after three months post-randomization. Data on insulin doses were available from study visits and intermediate telephone contacts and were therefore evaluated in 48 and 50 patients in the metformin and placebo groups, respectively. For other secondary outcomes, except for one drop-out patient (in the metformin group) having available data for LOCF, only patients who completed the study could be analyzed. Thus, other secondary outcomes were evaluated in a maximum of 48 and 45 patients in the metformin and placebo groups, respectively. During the trial, the routine method for measuring levels of folate was changed. Thus, for serum folate, only patients with baseline and end of treatment samples measured with identical such methods were evaluated (metformin: n = 29; placebo: n = 26). Otherwise, the screening and safety variables, reporting of adverse events and compliance were evaluated in all randomized patients exposed to the randomized study drugs (metformin n = 49; placebo: n = 51).

#### Numbers analyzed, ancillary outcomes

Except for the repeated measures HbA_1c_ analysis (see below), all analyses were performed as for the primary outcome (i.e., intention-to-treat). Follow-up HbA_1c_ data were available for a total of 99 patients (metformin: n = 48; placebo: n = 51) evaluated in the repeated measures HbA_1c_ analysis. Data from the analysis of the first and the last available HbA_1c_ is only shown for the subgroup of 72 patients (metformin: n = 35; placebo: n = 37) having available HbA_1c_ data from one to four months post-randomization.

### Primary outcome, primary analysis

After 12 months, the between-treatments difference in HbA_1c_ of 0.13% (−0.19; 0.44) was not significant (p = 0.422). In fact, the drop in HbA_1c_ (i.e., the change from baseline) was only significant in the placebo group by approximately 0.2% (Table 3).

**Table 3:** Metabolism-related variables in 100 patients with type-1 diabetes before and after one year of treatment with metformin or placebo.

	Baseline Metformin	Baseline Placebo	EOT Metformin	EOT Placebo	ΔMetformin	ΔPlacebo	ΔMetformin **versus** ΔPlacebo	**p-value**
***Subjects (number)***	**49**	**51**	**48**	**50**	**48**	**50**	**98**	
***HaemoglobinA_1c_*** * (%)*	**9.34** (0.92)	**9.35** (0.69)	**9.25** (0.94)	**9.12** (0.86)	−**0.10** (−0.32; 0.12)	−**0.23** (−0.45; −0.01)	**0.13** (−0.19; 0.44)	0.422
***Fasting plasma glucose*** * (mmol/l)*	**11.01** (4.59)	**13.30** (5.62)	**12.11** (4.60)	**11.72** (4.37)	**0.04** (−1.24; 1.32)	−**0.89** (−2.21; 0.43)	**0.93** (−0.93; 2.79)	0.324
***Total daily insulin dose*** * (Units)*	**59.7** (25.5)	**58.1** (17.9)	**56.8** (24.3)	**60.9** (20.0)	−**3.2** (−5.2 −1.2)	**2.5** (0.6; 4.5)	−**5.7** (−8.6; −2.9)	**<0.001**
***Total daily insulin dose*** * (Units/kg)*	**0.74** (0.29)	**0.74** (0.20)	**0.71** (0.25)	**0.77** (0.24)	−**0.03** (−0.06 −0.01)	**0.03** (−0.01; 0.06)	−**0.06** (−0.10; −0.02)	**0.003**
***Body weight*** * (kg)*	**80.23** (12.73)	**78.98** (14.98)	**78.78** (12.70)	**79.16** (16.63)	−**1.21** (−2.31; −0.12)	**0.53** (−0.60; 1.66)	−**1.74** (−3.32; −0.17)	**0.030**
***Body mass index*** * (kg/m^2^)*	**26.11** (3.55)	**25.78** (4.16)	**25.61** (3.39)	**25.85** (4.87)	−**0.37** (−0.72; −0.02)	**0.19** (−0.18; 0.55)	−**0.56** (−1.06; −0.05)	**0.031**
***Waist circumference*** * (cm)*	**93.29** (10.44)	**93.40** (12.10)	**92.58** (10.46)	**93.30** (12.88)	−**0.26** (−1.68; 1.15)	−**0.06** (−1.55; 1.44)	−**0.21** (−2.27; 1.86)	0.843
***Hip circumference*** * (cm)*	**100.31** (7.19)	**99.64** (10.32)	**98.44** (7.07)	**100.41** (10.62)	−**1.39** (−2.86; 0.07)	**1.51** (−0.04; 3.05)	−**2.90** (−5.03; −0.77)	**0.008**
***Waist/Hip ratio***	**0.93** (0.08)	**0.94** (0.07)	**0.94** (0.08)	**0.93** (0.07)	**0.01** (−0.01; 0.03)	−**0.01** (−0.03; 0.01)	**0.02** (−0.00; 0.05)	0.100

Data at baseline (0 month) and EOT (12 months) are raw absolute values given as mean (standard deviation). Data for ΔMetformin and ΔPlacebo represent the mean (95% confidence interval) estimated changes from baseline as predicted by the statistical model. The numbers of patients represent the maximum number of patients included in each analysis. Data are presented as intention-to-treat including last observation carried forward.

Abbreviations:

EOT: End of treatment.

### Primary outcome, secondary analyses

Adjustment for changes in total daily insulin dose did not change the conclusions for HbA_1c_. Also, for HbA_1c_, interactions by study drug treatment were not significant in subgroups of patients categorised by baseline levels of BMI, median baseline levels of HbA_1c_, total daily insulin dose, or by evidence of substantial changes in the insulin regimen during the trial (data not shown).

### Secondary outcomes, primary analysis

Levels of fasting plasma glucose showed approximately similar patterns to those for HbA_1c_ (Table 3). In contrast, after one year, significant differences were observed between the metformin and placebo groups of −5.7 units (−8.6; −2.9) in total daily insulin dose (p<0.001), of −1.74 kg (−3.32; −0.17) in body weight (p = 0.030) and of −2.90 cm (−5.03; −0.77) in hip circumference (p = 0.008). The changes in waist circumference and waist/hip ratio were not significantly different between treatments (Table 3).

### Adverse events and safety variables

The number of patients reporting minor or major episodes of hypoglycaemia was not significantly different between treatments (Table 4).

**Table 4:** Reported hypoglycaemic episodes in patients with type-1 diabetes during one year of treatment with metformin or placebo.

	Metformin (n = 49)	Placebo (n = 50)	p-value
*Minor hypoglycaemia:*			
*** Total number of patients with events*** * (%)*	**48** (98.0)	**49** (98.0)	−a
*** Total number of events during follow-up***	**5391**	**4752**	0.359
*** Number of events per subject month of follow-up, median*** * (range)*	**9.0** (0.0; 29.9)^b^	**6.4** (0.0; 22.4)^b^	0.312
*** Levels of plasma or blood glucose during events*** c	**2.87** (0.42)	**3.01** (0.58)	0.238
*Major hypoglycaemia:*			
*** Total number of patients with events*** * (%)*	**15** (30.6)	**10** (20.0)	0.254
*** Total number of events during follow-up***	**58**	**29**	0.261
*** Number of events per subject year of follow-up, median*** * (range)*	**0.0** (0.0; 10.8)^d^	**0.0** (0.0; 7.0)^d^	0.259
*** Levels of plasma or blood glucose during events*** c	**1.96** (0.50)	**2.06** (0.54)	0.508
*** Unconsciousness during events:***			
*** Total number of patients with events*** * (%)* ^g^	**6** (12.2)	**1** (2.0)	0.059
*** Total number of events during follow-up***	**10**	**2**	**<0.05** e
*** Number of events per subject year of follow-up, median*** * (range)*	**0.0** (0.0; 3.0)	**0.0** (0.0; 1.7)	**0.048**
*** Levels of plasma or blood glucose during events*** c	**1.78** (0.30)	**1.00** (−)	0.180

Data are included for at total of 99 patients (one patient, in the placebo group, out of a total of 100 randomized patients, had missing data on hypoglycaemia).

a Not compared statistically due to equal proportions of patients in treatment groups.

b **p<0.05** compared to enrolment (Table 2).

c Levels of circulating glucose were captured periodically by patients estimating average levels of blood/plasma glucose during hypoglycaemic events. The calibration (plasma or whole blood glucose) of hand held devices was not registrated, nor was devices calibrated at enrolment. Hence, data represent non-standardized plasma and/or blood glucose measurements. Not all patients had available glucose measurements during events. The total number of patients with available measurement was: Minor hypoglycaemia: metformin: n = 47; placebo: n = 49; Major hypoglycaemia: metformin: n = 11; placebo: n = 8.

d **p = 0.011** and p = 0.209 compared to enrolment for metformin and placebo, respectively (Table 2).

e p = 0.049888455.

More patients reported a metallic taste in their mouth with the metformin than with the placebo treatment (metformin: n = 7; placebo: n = 1; p = 0.029). Otherwise, no significant differences in the number of patients reporting new-onset gastrointestinal symptoms were observed between treatments either as separate symptoms (data not shown) or as any new-onset gastrointestinal symptom (metformin: n = 43; placebo: n = 39; p = 0.310).

Cardiovascular symptoms, infections and other complaints were infrequent and did not differ between treatment groups (data not shown). One non-hypoglycaemia-related SAE in each of six patients in the metformin group and 17 non-hypoglycaemia-related SAE's in 11 patients in the placebo group were recorded. These proportions of patients did not differ significantly between treatment groups (Table 5). Before unmasking, no non-hypoglycaemia-related SAE was considered potentially to have been associated with the study medication in the metformin group, whereas two such SAE's in the placebo group (two cases of diabetic ketoacidosis precipitated by gastroenteritis and myocardial infarction, respectively) were considered to be potentially related to the study medication. No cases of lactic acidosis occurred.

**Table 5:** Non-hypoglycaemia-related serious adverse events in 100 patients with type-1 diabetes allocated treatment with metformin or placebo for one year.

	Metformin (n = 49)	Placebo (n = 51)
***Serious adverse event type; No. of events [No. of patients with events]:***		
*** Psychiatric***	**0** [0]	**1** [1]
*** Neurological***	**1** [1]	**2** [2]
*** Cardiovascular***	**2** [2]	**5** [4]
*** Gynaecological***	**0** [0]	**1** [1]
*** Musculo-skeletal***	**1** [1]	**2** [2]
*** Metabolic***	**0** [0]	**2** [2]
*** Infections***	**1** [1]	**2** [2]
*** Trauma***	**1** [1]	**2** [1]
***Maximum number of events per patient***	**1**	**3**
***Total number of events [No. of patients with events]***	**6** [6]	**17** [11] a

a p = 0.151 and p = 0.287 between metformin versus placebo for the total number of events and total number of patients with events, respectively.

Levels of cobalamin and alkaline phosphatase decreased, whereas levels of plasma potassium increased significantly in the metformin group relative to the placebo group (mean [95% confidence intervals] difference metformin versus placebo in cobalamin: −83.3 pmol/l [−139.3; −27.3]; p = 0.004; alkaline phosphatase: −5.91 units/l [−10.77; −1.05]; p = 0.018; potassium: 0.20 mmol/l [0.02; 0.38]; p = 0.029). Levels of folate, haemoglobin, aspartate aminotransferase, sodium, creatinine and bicarbonate, as well as platelet and white blood cell counts did not significantly differ between treatments (data not shown). At end of treatment, in the metformin group, only one patient had levels of plasma potassium above 5.5 mmol/l (the patient had plasma potassium levels of 5.1 and 5.6 mmol/l before and after metformin treatment, respectively) not requiring treatment. The case of myocardial infarction and diabetic ketoacidosis, in the placebo group, also showed hyperkalaemia (potassium 7.7 mmol/l). Otherwise, no treatment of hyperkalaemia was necessary.

### Compliance and study drug dose

The median (range) compliance was 90.0% (33.0; 126.0) for the metformin group [n = 47] and 92.3% (42.5; 123.5) for the placebo group [n = 45] (p = 0.879). Approximately the same proportion of patients in these respective groups (34.7% [17/49] and 31.4% [16/51], p = 0.832) received a reduced study drug dose resulting in a median (range) daily study drug exposure of 1680 mg (416; 1954) in the metformin group and 1642 mg (152; 1989) in the placebo group (p = 0.836). Allocated treatments were correctly estimated at end of treatment by 57.4% (27/47) and 71.1% (32/45) of patients (p = 0.007) and by clinicians for 52.1% (25/48) and 64.4% (29/45) of patients in the metformin and placebo groups, respectively (p = 0.144).

### Ancillary analyses, HbA_1c_

During the run-in period, HbA_1c_ decreased in both treatment arms from approximately 9.5% at enrolment to about 9.3% at randomization (Tables 2 and 3), (p<0.05 compared to enrolment; p = 0.267 between treatment arms). Adjustment for the use of lipid-lowering therapy at enrolment/baseline, that is, statin and/or fish-oil therapies, did not change the conclusions for HbA_1c_ (data not shown). In the repeated measures HbA_1c_ analysis, the treatment by time interaction was significant (p = 0.032). Accordingly, after approximately three months, in the subgroup of 72 patients having available HbA_1c_ data from one to four months post-randomization, HbA_1c_ and insulin dose was significantly lowered with metformin compared to placebo with no significant differences in HbA_1c_ after 12 months (Table 6). Similar conclusions were obtained for all patients (data not shown).

**Table 6:** Effect of metformin versus placebo on follow-up (intermediate and study visit) levels of HbA_1c_ and insulin doses in 72 patients with type-1 diabetes during a one year period.

	Baseline Metformin	Baseline Placebo	Follow-up Metformin	Follow-up Placebo	ΔMetformin	ΔPlacebo	ΔMetformin **versus** ΔPlacebo	**p-value**
*Patients with available HbA_1c_ data from one to four months post-randomization:*								
***Subjects*** * (number)*	**35**	**37**	**35**	**37**	**35**	**37**	**72**	
***HbA_1c_, first available*** * (%)* a	**9.34** (1.02)	**9.32** (0.68)	**8.70** (1.01)	**9.18** (0.89)	−**0.64** (−0.86; −0.41)	−**0.14** (−0.36; 0.08)	−**0.50** (−0.81; −0.19)	***0.002***
***Time from randomization*** * (days)* b	−	−	**73** (39; 119)	**91** (32; 119)	−	−	−	*0.632*
***HbA_1c_, last available*** * (i.e., EOT) (%)* c	**9.34** (1.02)	**9.32** (0.68)	**9.07** (0.91)	**9.18** (0.79)	−**0.27** (−0.52; −0.02)	−**0.15** (−0.39; 0.10)	−**0.12** (−0.48; 0.23)	*0.494*
***Total daily insulin dose*** * (3 months)(Units)* d	**62.9** (28.2)	**55.7** (18.0)	**60.0** (25.8)	**56.1** (19.1)	−**2.7** (−4.3 −1.0)	**0.0** (−1.6; 1.7)	−**2.7** (−5.0 −0.3)	***0.026***

Follow-up HbA_1c_ data were obtained from planned study visits as well as from intermediate non-study outpatient clinic visits during the intervention period. All HbA_1c_ measurements obtained at least one month post-randomization were evaluated. Data are shown for the subgroup of 72 patients having available follow-up HbA_1c_ measurements from one to four months post-randomization. Data at baseline (0 month) and follow-up are raw absolute values given as mean (standard deviation) or median (range). Data for ΔMetformin and ΔPlacebo represent the mean (95% confidence interval) estimated changes from baseline as predicted by the statistical model.

a The first available follow-up HbA_1c_ measurement (in the group of 72 patients presented here, all the “first available” HbA_1c_ data were obtained at intermediate outpatient clinic visits). Given change from baseline estimates (i.e., ΔMetformin, ΔPlacebo and ΔMetformin versus ΔPlacebo) represent data without adjustment for time. Additional adjustment for time did not change conclusions substantially (data not shown).

b Time from randomization until the first available HbA_1c_ measurement.

c The last available follow-up HbA_1c_ measurement (obtained from study visits or intermediate outpatient clinic visits, that is, equal to EOT levels including last observation carried forward).

d Total daily insulin dose represents the last reported dose at the intermediate telephone consultations within the first three months post-randomization. The total insulin doses corrected for body weight (i.e. Units/kg) are not shown since data for body weight at the date of reporting insulin doses (i.e. at the intermediate telephone consultations) were not available.

Abbreviations:

EOT: End of treatment.

HbA_1c_: HaemoglobinA_1c_.

### Ancillary analyses, other

As compared to enrolment, both treatments significantly increased the number of minor hypoglycaemic events per month, whereas the number of major hypoglycaemic events per year was significantly increased in the metformin group only (Tables 2 and 4). Similar was observed when compared to the run-in period (data not shown). However, the reported number of minor and overall major hypoglycaemic events was not significantly different between treatments, whereas unconsciousness during major hypoglycaemic events was reported more frequently in the metformin group (Table 4). Similar was observed for the change in minor or overall major hypoglycaemic events versus enrolment or versus the run-in period between treatments (data not shown). The estimated average levels of blood/plasma glucose during events seemed to be approximately 1 mmol/l lower during major versus minor hypoglycaemia (not statistically tested) with no significant difference between treatments (Table 4). As estimated by the number of HbA_1c_ measurements, no significant difference between treatment groups was observed in the number of outpatient clinic visits (median [range] number of outpatient clinic visits per patient, in patients included in the intention-to-treat analysis, between randomization and end of treatment, metformin: 3 visits [0; 6]; placebo: 3 visits [0; 6], p = 0.362).

## Discussion

### Primary outcome, primary analysis

In this randomized, double-masked, parallel study, 100 patients with type-1 diabetes, preserved hypoglycaemic awareness and persistent inadequate glycaemic control were treated for 12 months with either metformin or placebo as an adjunct therapy to ongoing insulin therapy. After one year, there was no significant difference in the levels of HbA_1c_ between the two groups.

Of the previous studies investigating metformin treatment in patients with type-1 diabetes, and reporting efficacy data with respect to HbA_1c_ [16]–[24], that of Meyer et al. featured the largest sample and the longest intervention period (62 patients followed for six months). Patients presented with only mildly elevated pre-study levels of glycaemia (HbA_1c_ at enrolment was approximately 7.6%) and no significant glycaemic effect after metformin therapy was reported [20]. In comparison, our present study population presented with persistent and pronounced poor glycaemic control (mean HbA_1c_ at enrolment was approximately 9.5%). Thus, the two most extended trials of metformin treatment in patients with type-1 diabetes, that is, the study by Meyer et al and our present study, do not suggest any long-term glycaemic benefit of metformin therapy-no matter the pre-treatment level of glycaemic control.

### Primary outcome, secondary analyses

However, a number of studies have suggested improved glycaemic levels with metformin treatment of patients with type-1 diabetes [9], [12]–[14], [18], [19], [21]–[24]. These studies were of short duration (six months or less) and, in most cases, included specific subsets of patients selected because they presented potential glucometabolic disturbances, such as obesity [23] or insulin resistance [18], for example in adolescents [19], [21], [22]. In our study, we investigated adult patients with type-1 diabetes and poor glycaemic control with or without other known glucometabolic disturbances. Non-obese and overweight/obese patients were included and stratified according to their BMI. Baseline levels of HbA_1c_, insulin dose or BMI (i.e., suggested indices of glucometabolic disturbances potentially related to insulin-action) did not show statistical evidence of interaction on the glycaemic response to the two treatments. Hence, in our study, baseline variables, potentially related to insulin-action, did not seem to affect the long-term glycaemic response to metformin treatment. In agreement with most previous studies, we found significantly lower total daily insulin doses after metformin therapy than with the placebo group [9]–[13], [16]–[18], [20], [22]–[24]. The unaffected conclusions for HbA_1c_ after adjustment for changes in insulin-dose, suggest that other changes in, for example, life-style, could have masked a glucose-lowering effect of metformin therapy. Also, at similar glycaemic levels, the lower insulin-dose after metformin therapy, suggests a glucose-lowering and/or insulin-sensitizing effect of metformin therapy in patients with type-1 diabetes.

### Secondary outcomes, primary analyses

Body weight and hip circumference decreased significantly during metformin treatment relative to the placebo group. For body weight, similar has been observed during metformin therapy in obese and non-obese patients with type-2 diabetes [7], [27]. By contrast, except for one study [18], previous studies of patients with type-1 diabetes have reported no significant change in body weight after metformin therapy [17], [19]–[23]. In the general population, insulinaemia and adiposity have been demonstrated to be independent predictors of cardiovascular disease [28]–[31]. Hence, our present findings of decreased levels of such cardiovascular risk-markers (assuming total daily insulin-dose is a marker of insulinaemia) might translate into an improved cardiovascular outcome of metformin therapy in patients with type-1 diabetes and persistent poor glycaemic control.

In agreement with most [11], [20], [21], [23], but not all [22] previous studies, we found no significant difference between treatments in the incidence of minor or overall major hypoglycaemia. The proportions of patients with any new-onset gastrointestinal symptom were similar in the two treatment groups. Levels of cobalamin (but not of folate) and alkaline phosphatase were significantly lower and levels of plasma potassium was significantly higher during metformin treatment than with placebo, although this did not result in significant differences in the incidence of symptomatic complaints or in levels of haemoglobin, platelets or aspartate aminotransferase. No treatment of hyperkalaemia was needed in the metformin group. Thus, overall, metformin treatment was well tolerated in our study population. Compliance was satisfactory (≥90%) in both treatment groups.

### Ancillary analyses, HbA_1c_

Previous shorter-term studies (six months duration or less) proposed improved glycaemic control with metformin in patients with type-1 diabetes [9], [12]–[14], [18], [19], [21]–[24]. In our present study, the intermediate HbA_1c_ data suggested a time dependent effect including a transient lowering of HbA_1c_ after approximately three months in the metformin group. Hence, the discrepancies between studies, most likely, primarily related to differences in study lengths rather than differences in baseline glycaemic control, obesity etc.

### Ancillary analyses, other

More events of unconsciousness during hypoglycaemia were reported in the metformin group. Hence, although we recruited patients with preserved hypoglycaemic awareness and there was no significant difference in the incidence of reporting minor or overall major hypoglycaemia at enrolment or after one year, major hypoglycaemic events were potentially more serious with metformin than with placebo.

### Strenghts

In our opinion, the primary strength of the present study, besides being adequately powered, double-masked, randomized and with a clinical relevant primary outcome (HbA_1c_), is the length of the study (i.e., a one year treatment period). Such study duration is sufficient to overcome short-term changes in glycaemic control as well as to provide some evidence of potential longer-term effects. Moreover, the selected study population, that is, patients with longstanding poor glycaemic control, is important since these patients have the poorest prognosis and they might have shown glycaemic benefits from adjunct metformin treatment. Also, the setting of the study (i.e., to a large extent in the outpatient clinic) makes the results more relevant to the clinical practice compared to studies using only highly motivated scientific investigators for the follow-up of patients.

### Limitations

Our present study has limitations. First, except for gender, the clinical characteristics were not significantly different between those patients who agreed to participate compared to those who did not. After randomization, the proportions of men and women were similar between treatment groups. We therefore do not expect the recruitment process to substantially having affected the outcomes. Second, except for lipid-lowering therapies (i.e., statin and fish-oil therapies), at enrolment, treatment groups were well matched for other known clinical characteristics. We do not suspect the randomization procedure to have been inadequate and therefore can assume these observed imbalances to have been chance phenomena. Also, the similar conclusions for HbA_1c_ after adjustment for the differences in lipid-lowering therapies at enrolment/baseline suggest that these imbalances did not impact significantly on the glycaemic outcomes of the study. Third, we selected adult patients with a history of poor glycaemic control. Hence, although our findings agree with those of some shorter-duration studies [11], [16], [17], [20], we cannot rule out the possibility that other categories of patients with type-1 diabetes, for example, those with less severe glycaemic dysregulation, adolescents, overtly obese people, etc., might experience glycaemic benefits of metformin treatment during a follow-up of one year or more. Fourth, insulin doses were adjusted at the discretion of the patients and clinicians in the outpatient clinic. The adjustment for changes in insulin doses did not seem to significantly influence the overall result for HbA_1c_. Nevertheless, we cannot discount the possibility that increased attention to minimize or prevent the lowering of insulin doses would have lowered HbA_1c_ by treatment with metformin during one year. However, a treatment design aiming for minimal adjustments of insulin doses, could increase the risk and/or the severity of major hypoglycaemia (as suggested from our data) and so compromises its ethical legitimacy. Also, the interaction of changes in insulin treatment regimen and study drug treatment was not significant, the number of outpatient clinic visits did not differ significantly between treatment groups and only six patients or fewer started or stopped other concomitant medications during the study. Hence, except for potential lifestyle changes as outlined, we do not expect potential unintended differences occurring during the outpatient clinic follow-up to have substantially affected the primary outcome. Fifth, the analyses of intermediate HbA_1c_ data were not pre-specified. Also, the timing as well as the number of intermediate HbA_1c_ measurements was not standardized between patients. Hence, these data should be interpreted extremely cautious (i.e., they could be chance findings and hypothesis generating). Sixth, the estimated average blood/plasma glucose levels during hypoglycaemic events were self-reported and no calibration of blood/plasma glucose devices was done. Hence, the estimates of the absolute blood/plasma glucose levels during hypoglycaemic events were probably not accurate. However, the observed tendency towards lower reported blood/plasma glucose levels during major versus minor hypoglycaemia (although not statistically tested) agreed with our clinical expectation. Hence, we find it likely that these observed (potential) differences in blood/plasma glucose levels adequately reflected the corresponding differences in hypoglycaemic severity. Seventh, corrections for multiple testing were not performed. If p-values had been corrected according to the Bonferroni method, only total daily insulin-dose would have remained significant different between treatments (data not shown). However, the Bonferroni method, by nature, increases the risk of type II statistical errors. In our opinion, rather issues related to study-design as well as existing biological and clinical knowledge should judge the issue of multiplicity [32]. Most secondary and ancillary analyses were, as outlined, in agreement with previously demonstrated effects of metformin treatment in patients with type-1 or type-2 diabetes. We therefore do not expect multiplicity as a main driving force for our findings.

### Conclusions

In 100 patients with type-1 diabetes, preserved hypoglycaemic awareness and persistent poor glycaemic control, we observed no significant difference in glycaemic levels after one year in those with an adjunct treatment of metformin or of placebo. After one year, the incidence of overall hypoglycaemia was similar in the two treatment groups. Metformin therapy was well-tolerated and resulted in decreased total daily insulin doses, body-weight, hip-circumference and serum cobalamin as well as a clinically insignificant increase in plasma potassium. Hence, in our study population, metformin therapy did not offer a simple solution to a complex glucoregulatory problem. Further investigations into the potential cardiovascular-protective effects of metformin therapy in patients with type-1 diabetes are warranted.

## Supporting Information

Protocol S1 Protocol and Stastistcal analysis plan-english version. The study protocol and statistical analysis plan including all amendments prior to the breaking of the blind. English translation of the original (Danish) version.
(0.16 MB DOC)

Protocol S2 Protocol and statistical analysis plan-Danish version. The study protocol and statistical analysis plan including all amendments prior to breaking of the blind. The original Danish version.
(0.17 MB DOC)

Checklist S1 CONSORT Checklist
(0.10 MB DOC)

Amendment S1 Amendment september 2008 to the protocol and statistical analysis plan.
(0.06 MB DOC)
