# Supplementary material for: Effect of Adjunct Metformin Treatment in Patients with Type-1 Diabetes and Persistent Inadequate Glycaemic Control. A Randomized Study
Source: PLoS One. 2008 Oct 9;3(10):e3363. doi: 10.1371/journal.pone.0003363 (PMC2566605; doi:10.1371/journal.pone.0003363)
Supplement: Protocol S1 — Protocol and Stastistcal analysis plan-english version. The study protocol and statistical analysis plan including all amendments prior to the breaking of the blind. English translation of the original (Danish) version. (0.16 MB DOC) [file pone.0003363.s001.doc]

22.04.2008:

NOTE! This supplementary file represents an English translation of the original version of the study protocol as well as all subsequent amendments prior to unmasking of the study. Among the amendments, only the amendment specifically addressed to the Ethical committee, has been sent hereto for additional approval. Other amendments represent internal working documents for the carrying out of the study.

Date: 29.04.2003. Final version.

**Protocol ”Type-1-Metformin”**

Title:

Effect of Metformin on glycaemic control and non-glycaemic cardiovascular risk factors in patients with type-1 diabetes and persistent unacceptable regulation on treatment with insulin and diet.

Short title: Type-1-Metformin

Project group:

Søren Søgaard Lund, MD, Peter Jacobsen, MD, Minna Wittrup, MD, Lise Tarnow, senior scientist, MD, Merete Frandsen, Chief laboratory technician, Hans Henrik Parving, Chief Physician and Allan Vaag, Chief Physician, Steno Diabetes Center.

Responsible investigators:

Allan Vaag, Hans Henrik Parving.

Location:

Steno Diabetes Center, Niels Steensens Vej 2, DK – 2820 Gentofte, Danmark. Tlf. +45 3968 0800.

Background:

Increased mortality and morbidity among patients with diabetes is primarily caused by development of late diabetic complications (ref 1. 2). More than 90% of patients with type-1 diabetes will, with increasing diabetes duration, develop variant degrees of late diabetic complications (ref 1). Increased blood glucose is an important factor for development of all kinds of late diabetic complications (ref 1, ref 2). One of the most important ways to prevent the development and stop the progression of late diabetic complications is improved regulation/normalization of the blood glucose level, that is glycaemic control (ref 1, 3). The Diabetes Complication and Control Trial (DCCT) is the most extended study of the effect of improved glycaemic control in patients with type 1 diabetes. The study investigated approximately 1.500 patients followed for 6.5 years (ref 4) and showed that it was possible to reduce the risk of late diabetic complications with 35-90% by intensive blood glucose control. Moreover, it was shown that the risk for late diabetic complications was closely linked with the mean glycaemic control as estimated by HaemoglobinA1c (HbA1c). A later follow-up of the DCCT study have shown that the beneficial effect of intensive blood glucose control is persistent despite only small changes in the mean blood glucose level (0.4% in HbA1c) (ref 3). A crucial goal in the treatment of patients with type-1 diabetes is therefore to improve the glycaemic control without important adverse effects.

So far, the regulation of the blood glucose level in patients with type-1 diabetes have consisted of treatment with insulin and diet. In daily clinical practice this treatment is not sufficient to achieve the recommended level of glycaemic control (HbA1c below 7.5%) in approximately 60-70% of the patients at Steno Diabetes Center. The most recent data from the accreditation database at Steno Diabetes Center show that among patients with type-1 diabetes approximately 10% have a level of HbA1c greater than 12% (unpublished data). As yet, no other treatment options besides adjustment of insulin dose and diet exist.

Metformin is a drug that has been used in the treatment of type-2 diabetes through 30 years (ref.1). It exerts it’s effect by improving the insulin sensitivity in the muscle and the liver and by lowering the hepatic glucose production (ref.26). An important part of the cause of type-2 diabetes is decreased insulin sensitivity (insulin resistance). Metformin is the treatment of first choice in overweight patients with type 2 diabetes (ref.5). Treatment with Metformin and bed-time insulin in overweight patients with type-2 diabetes has shown improved efficacy in lowering the blood glucose level compared to insulin only (ref. 6-9).

Small studies of patients with type-1 diabetes without late diabetic complications (ref.10-13) and with incipient diabetic kidney disease (ref.14) suggest that also such patients, like patients with type-2 diabetes, are insulin resistant.

The effect of Metformin in patients with type-1 diabetes is poorly documented. A study of 10 patients with type-1 diabetes showed that Metformin increases insulin sensitivity in these patients (ref.15) while a recent published study of 27 adolescents with type-1 diabetes did not show effect of Metformin on the insulin sensitivity (ref.25). Other small studies have shown that Metformin has an insulin sparing effect, that is that the patients’ daily insulin requirement is reduced (ref.16-19, 24-25).

Two recent studies of Metformin treatment in patients with type-1 diabetes have shown conflicting results concerning the effect on glycaemic control. The first of these studies investigated the effect of Metformin treatment in patients with type-1 diabetes treated with continuous insulin infusions (insulin pump). The study investigated 62 adult patients (minimum age 40 years) having a minimum HbA1c at baseline of 7.58% - that is acceptable glycaemic regulation. The study did not show any significant effect on HbA1c by Metformin treatment after six months and no difference in the number of hypoglycaemic episodes (ref.24). The other study investigated 27 adolescents with type-1 diabetes and poor metabolic control (mean HbA1c at baseline was 9.3% and 8.6% in the Metformin and placebo group, respectably) and showed a significant absolute difference of 0.6% in Hba1c (Metformin versus placebo) after three months treatment. In the Metformin treated group, a tendency towards more mild hypoglycaemic episodes was observed. Major episodes of hypoglycaemia were observed in two cases in the Metformin group and one case in the placebo group (ref.25). Both studies confirmed, as outlined, previous studies results regarding the insulin sparing effect of Metformin

The previous studies of Metformin treatment of patients with type-1 diabetes have therefore, on a number of variables, shown conflicting results and have not had the necessary sample sizes and duration of intervention to conclude whether Metformin has a persistent beneficial effect on glycaemic control and hereby the long term prognosis in patients with type-1 diabetes (ref.9). It has therefore never been investigated whether treatment with Metformin, as adjunct therapy to insulin treatment, in patients with type-1 diabetes and poor metabolic regulation, improves the glycaemic control persistently, that is results in a decrease in HbA1c after a mininum of 12 months treatment.

Besides the potential beneficial effect of Metformin on glycaemic control also a number of other positive effects of Metformin have been reported. The United Kingdom Prospective Diabetes Study (UKPDS) showed that overweight patients with type 2 diabetes treated with Metformin had a significant lower risk of stroke, diabetes related events and total mortality compared with other treatments having a similar blood glucose lowering effect (ref.20). Other studies have shown that Metformin might lower lipidaemia (cholesterol and triglycerides) in patients with type-2 diabetes (ref.21). A suboptimal designed smaller study has shown that Metformin lowers blood pressure and urinary albumin excretion in 51 patients with type 2 diabetes and incipient diabetic kidney disease (microalbumunuria) (ref.22). These matters have not been investigated in patients with type-1 diabetes.

The small number of studies which have used Metformin in patients with type-1 diabetes found that the treatment was well tolerated (ref. 16,18,19,24,25).

If it is shown that Metformin can improve the glycaemic control and potentially have a beneficial effect on other risk factors it can mean a breakthrough in the treatment of patients with type-1 diabetes and poor metabolic control.

**Aim**

To investigate the effect of Metformin on glycaemic control and non-glycaemic cardiovascular risk factors in patients with type-1 diabetes and persistent poor glycaemic control.

**Population**

100 male and female patients with type-1 diabetes. The patients will primarily be recruited from Steno Diabetes Center and, moreover, from other endocrinology departments in the Copenhagen County and potentially by advertising.

**Inclusion criteria**

- Type-1 diabetes (WHO criteria) (ref.23)
- Age: > 18 years
- Age at onset < 35 years
- Diabetes duration > 5 years
- Fasting C-peptide < 0.3 pmol/l
- HbA1c > 8.5% during the last year

**Baseline characteristics**

Routine determination of variables related to the diabetic control and other specific endpoints (see later), diabetic late complications, aspartate aminotransferase (AST), factor II, VII,X, serum creatinine, electrocardiogram (ECG) , electrolytes, haemoglobin concentration, DNA for later pharmacogenetic subanalysis, serum cobalamin, erytrocyte folate, homocystein and other anthropometric and socioeconomic data (gender, age, height, body weight, fat distribution, ethnicity, smoking and physical activity, social class etc). At inclusion, other clinical and biochemical variables relating to the in- and exclusion criteria will be determined. The presence of potential late diabetic complications will be registered from patient records and by medical history. The patients opinion concerning the cause of the poor metabolic regulation (fear of hypoglycaemia, diet and injection compliance etc) will be registered. For women it will be registered whether they are pre- or post-menopausal. For pre-menopausal women, the use of adequate contraception (contraceptive pills, intrauterine device (IUD) or injected depot gestagen) will be ensured. Pre-menopausal women will have a pregnancy test (urinary human chorionic gonadotropin).

**Exclusion criteria**

- Kidney disease (serum creatinine above the upper normal limit for each gender).
- Liver disease (AST increased three times above the upper normal limit, alkaline phosphatase two times above the normal limit or factor 2-7-10 below the normal limit)
- Signs of congestive heart failure
- Serious organic or metabolic diseases, including cancer
- Pregnancy or planned pregnancy
- Abuse of alcohol or other abuse (narcotics)
- Hypoglycaemic unawareness

**Study protocol and design**

The study is designed as a single center, randomized, double-masked placebo controlled parallel study of Metformin as adjunct antiglycaemic treatment to ongoing insulin treatment over a period of 12 months. The observation period of 12 months is chosen to reach stable metabolic regulation since other studies, investigating the combination of Metformin and insulin in patients with type-2 diabetes, have shown that HbA1c do not stabilize until after 3-6 months (ref.8). The patients will be randomized to treatment with Metformin 500 mg which will be increased to 1 g x 2 daily before meal time or placebo on top of ongoing insulin treatment (the intervention period). The dose titration of the study drugs will take place with the minimum of one week interval. Starting dose of Metformin or placebo is 500 mg once daily increased by 500 mg per week to maximal 1 g 2 times daily. In case of adverse events considered related to the study medication the dose here off will be reduced. This will be the case for the active as well as placebo treatments since all health care providers are masked to treatments and therefore, any adjustments of the study medication dose has to be done assuming that all patients receive active treatment.

Half of the patients will be randomized to active treatment and the other half to placebo. Before initiating the intervention period there will be a run-in period of 1 month where all patients will be treated with placebo in a single-masked design. That is the patients will be told that they, from the start of the run-in period, will be treated with either Metformin or placebo. All patients will in fact be treated with placebo during the entire run-in period (single-masked). At baseline patients hereafter initiate the intervention period with Metformin or continue treatment with placebo. However, the patients will be randomized (double-masked) to the succeeding treatment type (Metformin or placebo) in the intervention period from the start of the run-in period. Hereby the time for starting the Metformin treatment should be masked to the patients - maximizing the placebo effect during the run-in period as well as in the intervention period. The health care providers will not be masked during the run-in period. The total follow-up time for each patient will be 13 months.

***Metformin 1g x 2***

***Placebo***

Run in

1st. visit

Inclusion

2nd. visit

Baseline.

1 month

12 months

***Placebo***

3rd. visit

4th. visit

5th. visit

6th. visit

There will be central randomization with block randomization with unequal block size and frequency. The investigators will not be aware of the randomization block size. Moreover, there will be a two step encrypting of the randomization code with treatment coded in first step as either A or B and second step as either Metformin or placebo. The patients will be stratified within these two treatment groups according to HbA1c and body mass index (BMI) in order to achieve equality of these variables between treatment groups.

The patients will, before initiation of the study drugs, receive instructions in the use of tablets by the study nurse and the titration of the dose will be by telephonic contact with the study nurse. Patients not being able to self-monitor blood glucose will moreover receive instructions in these matters. It is planned that the patients, during the run-in period and the last month of study, home-monitor 7 points profiles before and 1½ hour after each main meal and before bedtime two days per week. The patients’ blood glucose device will be calibrated before initiating the study. Patients who do not have their own blood glucose device will have the offer of borrowing a device at Steno Diabetes Center.

The patients will be encouraged to continue, as for as possible, their usual insulin treatment regimens both with respect to the dose, the timing, the blood glucose measurements (besides the last two weeks of the run-in period and the intervention period) as well as the injection techniques. In case of major hypoglycaemia inducing unconsciousness (NOTE: in Denmark termed “insulin chock”) or patients experiencing an unacceptable increase in the number of hypoglycaemic events (e.g nocturnal hypoglycaemia), adjustments in the insulin dose will be made based on an overall clinical judgement. In these cases, the insulin dose will be adjusted individually in corporation between the responsible medical doctors and nurses after the current local guidelines at Steno Diabetes Center. Other treatment modalities besides antiglycaemic medications will be continued or initiated according to good clinical practice during the intervention period. The type and dose of this medication will be registered at each visit during the study. The aim will be that this medication is kept unchanged during the study so that non vital need for adjustments in the medication will be postponed until after the study.

All patients will be encouraged to continue their usual lifestyle regarding diet and physical activity during the entire study period.

**Monitoring**

The primary endpoint of the study will be monitored immediately before the intervention period (baseline) and after 12 months.

In addition, during the study, the patients will have consultations by a doctor approximately every three to four months. At the inclusion and baseline visits (first and second visit) and at the completion of the study, the patients will have consultations by the medical doctors and nurses conducting the study. At each of these visits, HbA1c, blood glucose, body weight, blood pressure as well as measurement in three samples of 24-hour urinary albumin excretion rate will be monitored (along with other endpoints variables – see later). HbA1c will be measured in duplicate (i.e. a total of two analyses made on two different blood samples taken on the same day) at inclusion, baseline and at the study end. At other visits the HbA1c will be monitored once, that is a total of nine times per patient. At the visits at 3, 6 and 9 months after randomization the patients will have consultations with their usual medical doctor at the Steno Diabetes Center in the out-patient clinic at Steno Diabetes Center. These visits will therefore be performed according to standard routine procedures with respect to the consultation and the monitoring of variables in relation to the diabetic control (blood glucose, HbA1c, body weight, blood pressure, albuminuria, lipids, eye examinations etc).

In case of problems related to the study medication, patients as well as other health care providers at Steno Diabetes Center, will be able to contact the doctors and nurses conducting the study who will take further action. The patients will be provided with the telephone number to the study nurse. The patients are supposed to be in telephonic contact with the study nurse until the maximum adverse event-free dose of the study medication has been reached as well as in the weeks before termination of the study for the blood glucose measurements.

The compliance of the patients will be evaluated by tablet counting. Patients with known or foreseen compliance problems will be offered the use of a dosing box.

**Endpoints**

Primary:

- Change in HbA1c.

Secondary:

- Absolute HbA1c
- The number of documented plasma glucose concentrations below to 2.5 mmol/l during the whole study and in the last three months of the study
- Reported minor hypoglycaemia episodes and major hypoglycaemia episodes during the whole study and in the last three months
- Total insulin dose

The following secondary endpoints will be monitored at baseline and at the last visit at 12 months.

- Plasma-plasminogen activator inhibitor (PAI) antigen, PAI-activity, tissue-type plasminogen activator (t-PA)-antigen, t-PA activity, platelet-aggregation, platelet-number and release of serotonin from platelets after 10 minutes rest.
- Plasma fibrinogen
- Serum albumin
- Markers of endothelial function including Von Willebrand factor, intercellular adhesion molecules (ICAM), vascular cellular adhesion molecules (VCAM), selectin and endothelin
- Plasma-Homocystein
- Asymmetric DiMethylArginine (ADMA)
- Albumin excretion in three 24-hour urine collections
- Office blood pressure after 10 minutes rest
- Fasting lipids (total cholesterol, low density lipoprotein (LDL)-cholesterol, high-density lipoprotein (HDL)-cholesterol, very low density lipoprotein (VLDL)-cholesterol and triglycerides)
- Body weight, BMI, waist-hip ratio
- White blood cell count, C-reactive protein (CRP), Interleukin-6 (IL-6) and tumor necrosis factor-alpha.
- Serum creatinine, electrolytes, AST, alkaline phosphatase, factor 2-7-10, haemoglobin concentration

For all valuables the time of collection and analysis will be registered.

Extra blood samples will be collected for freezing in case of future need for extra analyses besides the once already mentioned.

**Duration:**

The study will be started approximately 1.st of October 2003 and inclusion of the patients will be expected to be finished approximately April 1, 2005. The study is therefore expected to be finished approximately first of April 2006, that is a total of 30 months.

**Usefulness of the investigation results**

It is expected that the results of the study can have a substantial impact for the treatment of patients with type-1 diabetes and poor metabolic regulation. Moreover, a positive effect on non-glycaemic cardiovascular risk factors, e.g. urinary albumin excretion rate, could reduce the risk for development of late diabetic complications especially cardiovascular disease and diabetic kidney disease.

**Data**

Source data will be not previously written or electronically registered data, with the exceptions of the registration of late diabetic complications which will be partly obtained from patients’ records.

Source data will be registered in the case report form (CRF) in electronically as well as in written form. The source data and the randomization code will be kept by Hans Henrik Parving/Allan Vaag and Søren Søgaard Lund at Steno Diabetes Center. The standard terms of the Danish Data Registry concerning security will be followed for personalized identifiable information and will be anonymised at the end of the study.

The randomization code will only be broken in case this is of vital importance of a patient’s safety and health. Only the person responsible for the study can decide about this. There will not be collected data after the study medication has been stopped.

There will be established a biobank with frozen samples (plasma, serum, urine and DNA material) for later analyses. These samples will be stored for an unlimited amount of time. All other samples will be destroyed immediately after analysis and latest at the end of the study.

**Safety evaluation**

The patients will, at all visits, be asked about any complications during the study and will be encouraged to report these by telephone to the investigators in between study visits. There will be considered three types of complications:

Adverse events: Any harmful unintended medical occurrence of the study drug no matter the dose.

Adverse drug reaction: Any unintended medical occurrence to a patient during the study period whether or not this is judged to be due to the study medication.

Serious adverse event or serious adverse drug reaction: Any untoward medical occurrence that at any dose: results in death, is life threatening requires in patient hospitalization or prolongation of existing hospitalization, results in persistence or significant disability/incapacity or is congenital anomaly/birth defect.

All adverse events and adverse drug reactions will be registered with type, time of occurrence, duration and any residual effects hereof or treatment and will be reported at the end of the study to the sponsors and to the Danish Medical Agency.

All serious adverse events or serious drug reactions will immediately be reported to the sponsors and the Danish Medical Agency.

There will not be registered any complications after the completion of the study.

Any complications during the study period will be diagnosed and treated in accordance with good clinical practice. The monitoring of the endpoints and baseline variables will contribute to prevent the occurrence of complications.

The estimated blood loss to asses the clinical variables during the investigations at baseline and after 12 months will be approximately 100 ml at each of the two occasions. At other study visits, the blood loss will be comparable to a standard routine visit in the outpatient clinic or at the general practitioner.

**Statistical considerations and power estimations**

Patients treated with placebo will be compared with patients treated with Metformin. The results will be expressed in mean and SD if the data is normally distributed otherwise as median (interquartile range (IQR)). For all variables there will be calculated changes during the study as well as absolute levels. For HbA1c all measured values during treatment will be included in the evaluations of the treatment effect. Other normally distributed variables will be compared by unpaired T-tests. Non-normally distributed variables will be compared by non-parametric tests.

Power Calculations:

To describe the necessary number of patients we have used the standard deviation (SD=1,0%) for HbA1c in 240 patients with type-1 diabetes, age less than 42 years, with HbA1c above 9.7% from Steno Diabetes Center.

The power calculation showed that at least 45 patients were needed in each group to show an absolute difference in HbA1c at 0.6% between the two treatment arms with alpha value equal to 5% (2-sided) and a beta equal to 20%.

Within an expected drop-out of approximately 10% it is therefore estimated that a total of 100 randomized patients will be needed. Any drop-outs after randomization will not be replaced.

**Ethical considerations**

The study will be conducted in accordance with the principles given in the Helsinki declaration after approval from the local ethical committee.

All participation in the study is voluntary. Consent for participating can, at any time before or during the study, be withdrawn by the patient.

The patient will receive oral as well as written information about the study before consent. The content of the oral information will be comparable to the written information and the time for the oral information will be agreed by the patient. The oral information will be given in unstressful physical surroundings by one of the medical doctors from the study. The oral information will be given after the written information and before inclusion in the study. The written information will be sent by a letter to the patient before the time of the oral information. The patient will be offered to bring an accompanying person along for the oral information and will be offered a minimum of 24 hours time for consideration between the oral information and consent. Besides the knowledge that the results from the study will provide about the optimal treatment of type-1 diabetes there will be no immediate advantages for the patients due to participating in the study.

All medication will be given free of charge to the patients during the study period. There will not be given any salary for participation in the study to the patients.

The inconvenience due to participation in the study will primarily relate to the adverse events of Metformin and insulin treatment, that is gastrointestinal symptoms, hypoglycaemia, rashes, headache and tiredness. Besides this, the time spending and the active participation in study visits, fasting, 24-hour urine sampling, blood pressure measuring, home blood glucose measuring and the inconvenience by blood sampling.

The health care providers in the investigator group are all employees at Steno Diabetes Center that is owned and partial administrated by Novo Nordisk A/S. The health care providers will not personally achieve any economically or professional advantages before, during or after the study.

Special qualified personal from the Ethical Committee or the Danish Medical Agency will, during the entire study period, have unlimited access to monitoring, auditing and inspection of source data, documents concerning the study participants including their patient records from Steno Diabetes Center.

Novo Nordisk A/S are entitled to withhold the data in a period of 4 months but can hereafter not withhold data from publication.

**Insurance**

The patients are covered by usual insurance from the medical products and from the patients insurance’s law.

**Publication and registration**

All the results from the study will be published in an international journal. The practical work in the study as well writing the report will predominantly be made by Søren Søgaard Lund with assistance from the other investigators. The study will be registered by the Danish Data Registry and the Danish Medical Agency.

**Carrying out of the study**

All the endpoints monitored during the study are implemented and are used in the daily routine at Steno Diabetes Center by the medical doctors from the study. The treatment regimens are used routinely in the daily clinical practice in other groups of patients at Steno Diabetes Center. The protocol will be available to the hospital leadership at Steno Diabetes Center for further clarification and insurance of the coverage of resources.

**Budget**

Investigating doctor 30 months circa 1.000.000 DKK

Half laboratory technician 30 months circa 500.000 DKK

Half nurse 30 months circa 500.000 DKK

Biochemical analyses etc circa 200.000 DKK

Transportation of patients circa 100.000 DKK

Package and labeling of tablets circa 50.000 DKK

Total 2.350.000 DKK

In addition to this there will be expenses to the study medication during 12 months:

Metformin-tablets: 4 tablets daily per patient x (366 days + extra 31 days) = 1.588 tablets per patient = 16 containers with each 100 tablets per patients = 1.600 x (50 patients + extra 5 patients) = 88.000 tablets (the same amount of placebo tablets will be used = 88.000 tablets Metformin placebo). For the run-in period a calculated placebo tablet dose of 1 tablet daily per patient x (31 days + extra 31 days) = 62 tablets per patient = 1 container with each 100 tablets per patient = 100 tablets x (100 patients + 50 extra patients) = 15.000 tablets. The total number of Metformin placebo tablets will be 15.000 + 88.000 tablets = 103.000 tablets.

In the above calculations an extra 31 days treatment is included for both the run-in period and intervention period in order to make the visit date more flexible for the patients and health care providers. Also, an extra 50 patients are calculated for the run-in period since it is expected that a number of patients will drop out during the run-in period due to e.g. non-compliance. Since all patients are randomized to a study drug number from the start of the run-in period, the extra number of placebo tablets will enable that one study drug number can be used more than once from those patients who have dropped out during the run-in period. By this procedure it is avoided that patients who drop-out from the run-in period will block a study drug number for the intervention period. In the intervention period study medication for an extra 10 randomized patients has been calculated (5 extra Metformin + 5 extra Metformin placebo). This medication is to be used in the occurrence of patients who lose substantial parts of the study medication or will be unable to give visit at scheduled times as well as for quality controls of the packaging procedure at KLIFO.

The expenses to the salary for the doctor in 30 months (DKK 1.000.000) and half nurse in 30 months (DKK 500.000) are covered via the budget in other ongoing studies at Steno Diabetes Center. The Danish Diabetes Association has moreover granted the study by DKK 100.000 provided that the study will be approved by the ethical committee. The remaining part of the budget are not yet covered for that is DKK 2.350.000 minus DKK 1.500.000 minus DKK 100.000 = **DKK 750.000.**

GEA A/S has provided support for the study by delivering Metformin tablets and comparable placebo.

Patients on insulin treatment are entitled to test materials free of charge due to the social service law, paragraph no. 97.

During the study there will be applied for funding from the clinical development foundation at Steno Diabetes Center and from other relevant community foundations as well as from the medical industry.

References:

(1) Medicinsk Kompendium. 15 udgave ed. Nyt Nordisk Forlag, Arnold Busck, 2000.

(2) Parving H-H, Østerby R, Ritz E. Diabetic nephropathy. In: Brenner BM, Levine S, editors. The Kidney. Philadelphia: WB Saunders, 2000: 1731-1773.

(3) Effect of intensive therapy on the microvascular complications of type 1 diabetes mellitus. JAMA 2002; 287(19):2563-2569.

(4) The effect of intensive treatment of diabetes on the development and progression of long-term complications in insulin-dependent diabetes mellitus. The Diabetes Control and Complications Trial Research Group. N Engl J Med 1993; 329(14):977-986.

(5) Type 2-diabetes og det metaboliske syndrom - diagnostik og behandling. Ugeskr Læger 2000; Klaringsrapport nr. 6.

(6) Giugliano D, Quatraro A, Consoli G, Minei A, Ceriello A, De Rosa N et al. Metformin for obese, insulin-treated diabetic patients: improvement in glycaemic control and reduction of metabolic risk factors. Eur J Clin Pharmacol 1993; 44(2):107-112.

(7) Robinson AC, Burke J, Robinson S, Johnston DG, Elkeles RS. The effects of metformin on glycemic control and serum lipids in insulin-treated NIDDM patients with suboptimal metabolic control. Diabetes Care 1998; 21(5):701-705.

(8) Yki-Jarvinen H, Ryysy L, Nikkila K, Tulokas T, Vanamo R, Heikkila M. Comparison of bedtime insulin regimens in patients with type 2 diabetes mellitus. A randomized, controlled trial. Ann Intern Med 1999; 130(5):389-396.

(9) Daniel JR, Hagmeyer KO. Metformin and insulin: is there a role for combination therapy? Ann Pharmacother 1997; 31(4):474-480.

(10) Lager I, Lonnroth P, von Schenck H, Smith U. Reversal of insulin resistance in type I diabetes after treatment with continuous subcutaneous insulin infusion. Br Med J (Clin Res Ed) 1983; 287(6406):1661-1664.

(11) Lonnroth P, Blohme G, Lager I, Tisell LE, Smith U. Insulin resistance in fat cells from insulin-treated type I diabetic individuals. Diabetes Care 1983; 6(6):586-590.

(12) Yki-Jarvinen H, Koivisto VA. Insulin resistance in type I diabetes: prevalence, pathogenesis and therapeutic approaches. Ann Clin Res 1984; 16(2):74-83.

(13) Ekstrand AV, Groop PH, Gronhagen-Riska C. Insulin resistance precedes microalbuminuria in patients with insulin- dependent diabetes mellitus. Nephrol Dial Transplant 1998; 13(12):3079-3083.

(14) Yip J, Mattock MB, Morocutti A, Sethi M, Trevisan R, Viberti G. Insulin resistance in insulin-dependent diabetic patients with microalbuminuria. Lancet 1993; 342(8876):883-887.

(15) Gin H, Messerchmitt C, Brottier E, Aubertin J. Metformin improved insulin resistance in type I, insulin-dependent, diabetic patients. Metabolism 1985; 34(10):923-925.

(16) Gin H, Slama G, Weissbrodt P, Poynard T, Vexiau P, Klein JC et al. Metformin reduces post-prandial insulin needs in type I (insulin- dependent) diabetic patients: assessment by the artificial pancreas. Diabetologia 1982; 23(1):34-36.

(17) Janssen M, Rillaerts E, De L, I. Effects of metformin on haemorheology, lipid parameters and insulin resistance in insulin-dependent diabetic patients (IDDM). Biomed Pharmacother 1991; 45(8):363-367.

(18) Pagano G, Tagliaferro V, Carta Q, Caselle MT, Bozzo C, Vitelli F et al. Metformin reduces insulin requirement in Type 1 (insulin-dependent) diabetes. Diabetologia 1983; 24(5):351-354.

(19) Schatz H, Winkler G, Jonatha EM, Pfeiffer EF. Studies on juvenile--type diabetes in children. Assessment of control under treatment with constant and variable doses of insulin with or without addition of biguanides. Diabete Metab 1975; 1(4):211-220.

(20) Effect of intensive blood-glucose control with metformin on complications in overweight patients with type 2 diabetes (UKPDS 34). UK Prospective Diabetes Study (UKPDS) Group. Lancet 1998; 352(9131):854-865.

(21) Wulffele MG, Kooy A, De Zeeuw D, Stehouwer CD, Gansevoort RT. The effect of metformin on blood pressure, plasma cholesterol and triglycerides in type 2 diabetes mellitus; a systematic review. Br J Clin Pharmacol 2002; 53(5):549P-550P.

(22) Amador-Licona N, Guizar-Mendoza J, Vargas E, Sanchez-Camargo G, Zamora-Mata L. The short-term effect of a switch from glibenclamide to metformin on blood pressure and microalbuminuria in patients with type 2 diabetes mellitus. Arch Med Res 2000; 31(6):571-575.

(23) WHO Report Part 1: Diagnosis and Classification of Diabetes Mellitus 1999.

(24) Meyer L et al: The Benefits of Metformin Therapy During Continuous Subcutaneos Insulin Infusion Treatment of Type 1 Diabetic Patients. Diabetes Care 25: 2153-2158, 2002.

(25) Hamilton J et al: Metformin as an Adjunct Therapy in Adolescents With Type 1 Diabetes and Insulin Resistance. Diabetes Care 26: 138-143, 2003

(26) Dunn J and Peters D.H.: Metformin. Drugs 1995, 49 (5): 725-729.

**Date: 27.06.2003.**

**Internal amendment to the protocol ”Effect of Metformin on glycaemic control and non-glycaemic cardiovascular risk factors in patients with type-1 diabetes and persistent unacceptable regulation on treatment with insulin and diet.“. Short running title “Type-1 Metformin”.**

The numbers will refer to the ICH Guideline in GCP.

Ad 6.1.1:

Date: April 29, 2003. Protocol code: KA 03046gs

Ad 6.1.3:

Sponsor (delivery of study medication):

GEA Ltd. Kanalholmen 8-12, 2650 Hvidovre, Denmark. Reference-person: Mette Elbæk.

Sponsor investigator:

Steno Diabetes Center

Niels Steensens Vej 2

DK - 2820 Gentofte

Denmark

Reference persons: Chief Physician Allan Vaag and Chief Physician Hans-Henrik Parving.

Ad 6.1.4.

Responsible for the investigation:

Chief Physician Allan Vaag, Phone number: +45 3075 0234

The responsibility will be the following for the participation in the project group:

Chief Physician Allan Vaag and Hans-Henrik Parving: overall responsible for carrying out the project, the patients treatment and observance of the ethical rules.

The medical doctors Lise Tarnow, Minna Wittrup, Peter Jacobsen and Søren Søgaard Lund:

Responsible for the practical carrying out of the project, including recruitment of patients and treatment, study drug handling, data collection and analysis/reporting.

Chief laboratory technician Merete Frandsen:

Senior responsible for the biochemical analysis of blood, urine and other tissue samples.

The laboratory technician Ulla Meng Schmidt, Birgitte Vilsbøl Hansen, Berit Ruud Jensen, Lotte Pietraszek, Tina Ragnholm Juul and Ingelise Rossing:

Responsible for the sample collections and treatment of blood, urine, tissue samples for biochemical analysis.

Nurse Bente Blaaholm Nielsen:

Together with the study doctors responsible for the carrying out the study, patient recruitment, treatment and handling of the study medication.

Secretary Ingelise Holstein:

Central randomization, that is allocation of patients to treatment arms.

Ad 6.1.7. See point 6.1.3.

Ad 6.1. (Summary)

Aim:

To investigate the effect of Metformin on the glycaemic regulation and other cardiovascular risk factors in patients with type-1 diabetes with persistent inadequate regulation on treatment with insulin and diet.

**Background:**

90% of patients with type-1 diabetes will develop late diabetic complications, that is micro-and macrovascular disease and neuropathy. Poor glycaemic regulation is an important risk factor for development of these complications. The DCCT-study showed that improved glycaemic regulation can prevent or delay progression in these late complications. So far, insulin treatment and diet has been the only options for improving the glycaemic regulation in patients with type-1 diabetes. Nevertheless, a substantial part among these patients have persistent poor glycaemic regulation despite intensive insulin treatment.

The drug Metformin has been used in patients with type-2 diabetes and has shown to improve the glycaemic regulation in combination with insulin as well as lowering the risk for development of macrovascular complications. Only few and small studies have investigated Metformin treatment in patients with type-1 diabetes. These studies have suggested a beneficial effect of Metformin in this category of patients.

**Method:**

100 patients with type-1 diabetes and persistent poor glycaemic regulation, that is HbA1c > 8.5%. The first month, all patients are treated with placebo. Hereafter, half of the patients are treated with metformin and the other half continues placebo treatment during 12 months. All patients continue the ongoing insulin treatment unchanged. Before and after the initiation of adjunct Metformin treatment, the glycaemic regulation and the effect of other known cardiovascular risk factors, that is blood pressure, lipidemia, urinary albumin excretion rate etc will be evaluated.

**Utility:**

The study will show whether Metformin treatment can improve the glycaemic regulation and hereby the prognosis in patients with type-1 diabetes with persistent poor glycaemic control despite intensive insulin treatment. Due to poor glycaemic control this group of patients carry an especially high risk for development of invalidating diseases in a number of organs and hereby reduced quality of life and shortening of life.

Ad 6.2.5.

The study will be conducted in accordance with the good clinical practice (GCP) guidelines as far as possible. Since the study already has been registered and approved by the Danish Data Registry, the ethical committee as well as the Danish Medical Agency as a non-GCP monitored study there will be made supplementary internal documents to guide the carrying out of the study in accordance with the GCP guidelines the best as possible. These guidelines will be in accordance with the already approved protocol and with the GCP guidelines. However the patient information will be available to the ethical committee for re-evaluation of whether the GCP unit at the Copenhagen University can be approved to monitor personalized data from the study.

Ad 6.4.2.

Type of study and design: Single centre, double-masked randomized placebo controlled parallel study.

Ad 6.4.3.

There will be used central randomization and block randomization with unequal block size and frequency. The investigators will not know the randomization block size. There will be two step encrypting of the randomization code with the treatment coded in the first step as A or B and in the second step as Metformin or Placebo. The patients will be stratified into treatment groups according to HbA1c (greater or smaller than 9.5% and BMI) (greater or smaller than 25 kg/m^2^) to achieve an equal distribution of these variables between the two treatment arms. The central randomization will be carried out at Steno Diabetes Center by an employee without other participation in the study. The allocations of patients to the two treatment groups will be carried out by pre-specified criteria for HbA1c and BMI respectively.

Ad 6.4.6.

Patients cannot continue the study in case of serious unexpected adverse drug events (as defined in the protocol - page 11 in the present document, but page 9 in the original document) with relation to the study medication. An exception to this is any occurrence of hypoglycaemia since this is not considered unexpected. The study will be stopped in case of information showing that the risk of completing the study is unacceptable high or if other studies show that treatment with Metformin as adjunct therapy to insulin is better that insulin treatment alone.

Ad 6.5.2.

The term heart failure refers to patients classified as having heart failure, New York Heart Association (NYHA) class I-II with ejection fraction below 30%, NYHA class III-IV or in case of findings indicating heart failure, that is oedema of cardiac origin or stasis/3rd. stethoscopic heart sound.

The term serious organic or metabolic diseases including cancer includes diseases that are expected to be life shortening to the patients expected lifetime or, to reduce the well being of the patients to a degree that can influence the course of the study or, where experimental treatment are contra-indicated for ethical reasons, for example ongoing cancer or schizophrenia. Patients with a previous cancer diagnose where the treatment have resulted in patients considered as cured (that is the patients expected lifetime is not reduced) can participate in the study.

Ad 6.5.3.

Withdrawal criteria:

These will be identical to the exclusion criteria, see the protocol (page 5 in the present document, but page 4 in the original document).

Ad 6.5.3.

Data for drop-outs:

Drop-outs will be included in an intention to treat analysis.

Ad 6.6.

About study drug number: Further information will follow.

Ad 6.9.5.

Missing data: Further information will follow about this.

**Miscellaneous:**

Hypoglycaemia with or without symptoms are considered an expected adverse event to the treatment and will therefore not be reported (to the sponsors etc.) as an adverse event unless there has been need for in-patient hospitalization. Hypoglycaemia will be registered in the CRF similar to other adverse events.

Doctors that monitor the patients during the study will all receive instructions in asking for adverse events, other medications and compliance with the study medication even though it is the patients’ usual doctor at the Steno Diabetes Center.

Patients from outside Steno Diabetes Center will have a file at the Steno Diabetes Center. There will be made a DNA bio bank for later pharmacogenetic analysis.

Advertising suggestions have not been made yet and wait further decision from the project group to whether patients will be recruited by this method.

Patient information and informed consent from the ethical committee are available at this moment.

**Date: 05.07.2004.**

To the Ethical Committee

Stationsparken 27

DK – 2600 Glostrup

Danmark

Amendment to the protocol KA 03046GS

Project title: “The effect of Metformin on glycaemic control and non-glycaemic cardiovascular risk factors in patients with type-1 diabetes and persistent unacceptable regulation on treatment with insulin and diet.”. Short running title: “Type-1 Metformin”.

In the protocol the following will be changed:

Page 7 line 6 from the bottom: It is planned that the patients, during the run-in period and the last month of study, home-monitor 7 points profiles before and 1½ hour after each main meal and before bedtime two days per week. This part will be omitted.

Page 8 line 3 from the top: The following will be omitted: “(besides the last two weeks of the run-in period and at the end of the study)”.

22.04.2008: NOTE! The page and line numbers given above have been changed to match the present document.

**Date: 27.08.2004.**

**Internal amendment number 2.**

**To the study with the title: ”Effect of Metformin on glycaemic control and non-glycaemic cardiovascular risk factors in patients with type-1 diabetes and persistent unacceptable regulation on treatment with insulin and diet.“ Short running title: “Type-1 Metformin”.**

**Ad Short running title:**

The short running title is changed in these internal documents to Met-1.

**Ad randomization procedure:**

The randomization procedure is changed in the following way: At inclusion in the study, at the first visit, the patients are given a patient number that follows the patient throughout the entire study period. This number will be written on the study medication in the run-in period (the medical containers with the red etiquette). At the second visit (baseline) after approximately 1 month, the randomization will be performed according to the previous described procedure. At the randomization, the patients will additionally to the patient number be given a three digit study drug number. This study drug number will be pre-printed on all randomized study drug containers (medicine containers with the white etiquette) and there shall therefore not be written any further details on these containers. Thus, the patients are given the randomization number after the run-in period has been completed which is in contrast to what was described in the previous version of the protocol where the patients, from the start of the run-in period, was given the randomization number. As previously planned, the patients will still receive information that they initiate the study drug from the beginning of the run-in period whereby the time for initiation of the active and placebo treatment will remain masked for the patients.

**Ad stratification**

The stratification procedure will be carried out with consecutive numbers (that is patients who are to be randomized will be given the next available consecutive study-drug number in increasing respectively decreasing order according to the table below) in four equally sized groups according to baseline levels of HbA1c and BMI. The four groups are the following:

1. HbA1c ≥ 9.6% and BMI ≥ 25 kg/m^2^ will be given study drug number 1-25 (starting from 1,2,3 etc)
2. HbA1c ≥ 9.6% and BMI < 25 kg/m^2^ will be given study drug number 26-50 (starting from 50,49,48 etc)
3. HbA1c < 9.6% and BMI ≥ 25 kg/m^2^ will be given study drug number 51-75 (starting from 51,52,53 etc)
4. HbA1c < 9.6% and BMI < 25 kg/m2 will be given study drug number 100-76 (starting from 100,99,98 etc)

In case of unequal distribution of patients in the four groups, the groups can be combined. Primarily will the group 1 and 2 be combined and/or group 3 or 4 depending on which of the four groups have used all study drug numbers. In case two groups will be combined, the same procedure for allocating consecutive numbers will be used unchanged in increasing or decreasing order, respectively, in any given group. If, for example, group 1 and 2 are combined, due to all study numbers in group 1 have been used, then the next patient, who is to have a study drug number in group 1, will be moved to group 2, but will still be given a study drug number starting from below, that is from number 26,27,28 and so on, and similarly for other group combinations.

In case of a group being completed which have already been combined (e.g. group 1 and 2 and group 3 og 4) and there still are available study drug numbers in the other two groups, then these two remaining groups will be combined and hereafter the patients will be given a study drug number according only to the HbA1c value and not to the BMI value. The rationale herefore is that it is considered more important to distribute the HbA1c equally between the two treatment arms than the BMI. The HbA1c level will continue to divide at < 9.6%/≥ 9.6% and, in the combined group, the one of the two previous BMI groups with most number of randomized patients will continue to be randomized in consecutive increasing or decreasing order, respectively, in patients belonging to that HbA1c group. The other HbA1c group will then start by numbers from the opposite end of the combined group. In case an equally number of patients have been randomized in each group, then the patient will be given the lowest available study drug number and patients from the other HbA1c group will start/continue from the opposite end of the combined group. If, for example, group 3 and 4 are combined and there are, so far, randomized 7 patients in group 3 and 15 patients in group 4 then patients with HbA1c < 9.6% will be given study drug numbers starting from the highest number in group 4 independent of the BMI and the patients with HbA1c ≥ 9.6% will be given study drug numbers starting from the lowest number in group 3 also independent of BMI and so on. In contrast, if there were randomized equal numbers of patients in group 3 and 4 then the next patients will be given numbers from study group 3 and patients from the opposite HbA1c group will be given study drug numbers from group 4.

**Ad reporting:**

At the reporting of the study in a scientific journal or equally, then Søren Søgaard Lund will be the first named author on all publications unless other written agreement among the involved authors have been made.

**Ad in-/exclusion criteria:**

In the protocol as well as its previous amendments and the CRF there have been a typographical error meaning that the sign for less than or equal to (≤) respectively greater than or equal to (≥) have been replaced by only less than (<) or greater than (>) respectively. Therefore, in all in- and exclusion criteria the sign less than (<) and greater than (>) should be replaced by less than or equal to (≤) and greater than or equal to (≥), respectively. However the exclusion criteria for creatinine will remain greater than 110 micromol/l for women and greater than 130 micromol/l for men, AST will remain greater than 150 U/l and factor II,VII and X will remain less than 0.7 as stated in the CRF, otherwise there are no exceptions to the replacements. Therefore the inclusion criteria of, for example, HbA1c is ≥ 8.5% during the last year and so on.

**Ad study visit:**

As stated in the protocol the health care of the patients after initiation of the randomized study medication will take place in the outpatient clinic at Steno Diabetes Center after usual procedures except for the end-of-study visits which will take place in the research department (department 520) at Steno Diabetes Center. The registration of adverse events to the study drugs and the treatment hereof with respect to the adjustment of the study medication and/or insulin will, however, primarily be taken care of through telephonic contact by the doctors or nurses from the study. There will be at least three planned telephone consultation scheduled equally during the study period per patient. Also the registration of adverse events can be carried out by the patients completing a questionnaire which will be evaluated by the nurses and doctors from the studies for any needs of treatment. In case of extra need for health care this can be made in the outpatient clinic or at the research department at Steno Diabetes Center. The visit in the outpatient clinic will not be filed in the CRF. The information in the CRF will be collected by telephonic contact or questionnaire as mentioned.

**Ad monitoring of HbA1c:**

In the protocol it is stated that HbA1c will be monitored 9 times per patient during the study. Since the monitoring of HbA1c during this study between randomization and end of study will be during the routine visit in the out-patient clinic, the number of HbA1c measurements will depend on the number of these visits and will therefore differ between patients. It is not the plan that these HbA1c measurements from the outpatient clinic are to be included in the evaluation of the efficacy except for patients dropping out during the randomized period. For these patients, the HbA1c measurements from the outpatient clinic can be evaluated with the last observed value as the measure of efficacy.

**Ad blood glucose device:**

In the protocol it is stated that patient’s own blood glucose device will be calibrated prior to the study. Since the systematic blood glucose measurements have been omitted as an endpoint in the study - according to a previous protocol amendment - it is considered that there are no longer need to do this. The patients will, however, after usual procedure at Steno Diabetes Center be offered calibration of the blood glucose device in case of need for this.

**Ad compliance:**

After the run-in period, the patients use of tablets will be registered. Compliance with the study drug in this period will not be a criterion for continued participation in the study e.g. the randomized period.

**Date: 27.10.2004.**

**Internal amendment number 3.**

**To the study with the title: ”Effect of Metformin on glycaemic control and non-glycaemic cardiovascular risk factors in patients with type-1 diabetes and persistent unacceptable regulation on treatment with insulin and diet.“ Short running title: “Type-1 Metformin”.**

**Ad fasting C-peptide:**

As otherwise stated in the protocol, there will not, in all patients, be measured a fasting C-peptide before inclusion or randomization. Instead a non-fasting C-peptide will be measured at the inclusion visit (visit 1) with identical cut-off limits as for the fasting C-peptide. In case this value is greater than or equal to 300 pmol/l, there will be performed supplementary test of the beta cell function for example fasting C-peptide or a glucagon test before the patient can be randomized. Only in case these measurements suggest type-1 diabetes can the patient be randomized.

Søren Lund 27/10, 2004

Allan Vaag 27/10,2004

**Date: 27.12.2004.**

**4.th Addendum**

**Protocol: Met-1**

**Sponsor-investigator/medical responsible: Allan Vaag.**

**Ad inclusion criteria:**

HbA1c ≥ 8.5% during the last year – this means that the mean of the HbA1c measurements must be ≥ 8.5%.

Moreover, the HbA1c at visit 1 (enrolment) must be ≥ 8.5% - and at visit 2 (randomization) ≥ 8.0%.

**Ad hepatic biochemical variables:**

Due to an error in procedures, not all patients have had a measurement of alkaline phosphatase at enrolment. Since this variable is of lesser clinical importance, patients with missing values hereof at enrolment will remain in the study. There will be a note in the CRF regarding the clinical relevance of missing values. From now on, the practical procedure for requesting biochemical analyses will be changed in order to ensure that all patients have a measurement of alkaline phosphatase at enrolment.

Factor II, VII, X is of lesser clinical importance for the eligibility of the patient in the study and deviations judged as being not clinical relevant or missing values will not have any consequence for the patients participation in the study. There will be a note in the CRF regarding the clinical relevance of missing values.

**Ad screening period:**

This has to be a minimum of 25 days and a maximum of 100 days.

**Wittness the signature of this document:**

Sponsor-investigator/medical responsible:

Name:_______________; Signature:_________________; Date:____________

**Date: 22.12.2006.**

**Met-1 analysis plan (statistical analyses)**

**According to the protocol by from Søren Søgaard Lund and inspired by K Hermansen Diabetes Care 29: 1269,2006.**

Randomized study of the effect of Metformin as adjunct therapy to ongoing insulin treatment in patients with type-1 diabetes, 100 randomized and followed up for one year. Primary analysis intention to treat, last HbA1c value and insulin dose, at least three month after randomization are used as endpoint (last value carry forward) if the patients drop out before 12 months. If the patient drop out prior to this, then the HbA1c cannot be evaluated meaningfully. A per protocol analysis can be made explorative in case of drop out to evaluate the treatment potential, but this will be secondary to the intention to treat analysis.

1. Demographic description of the two randomized groups with respect to age, gender, duration of diabetes, HbA1c, BMI, insulin dose per kg body-weight, retinopathy, urinary albumin excretion category. Drop outs.
2. Analysis of the primary endpoint, effect of Metformin on HbA1c and analysis of variance (ANOVA) adjusted for baseline HbA1c (the change in HbA1c from baseline in the two groups) with treatment as fixed effect. If randomization has made substantial differences in parameters (especially BMI) according to point no. 1 this will be adjusted for in the analysis. It will be evaluated individually for each parameter if such difference is substantial.
3. If the primary analysis is negative, then the number and the interpretation of secondary analyses will be limited. No matter, however, an analysis as under point no. 2 will be made adjusted for BMI as well as an analysis stratified by the median HbA1c and BMI minor or greater than 25.
4. Other secondary analyses:
   - as point no. 2 and 3 adjusted for insulin resistance, insulin per kg, waist-hip ratio, eGDR (according to T Orchard) as well gender, age and diabetes duration and change in insulin dose from the beginning to the end (potentially fasting blood glucose, C-peptide, potentially stratified according to GAD antibodies in case increased BMI will increase the effect in order to exclude type 2 diabetes, especially if C-peptide is not conclusively negative).
   - subgroup analysis of patients who, during the study period, change insulin treatment regimen, for example, from human insulin to insulin analogues and/or changes the number of daily injections, initiate continues insulin infusions (pumps) or similar substantial changes in the treatment regimen (changes in insulin dose are not considered as a substantial change in the insulin regimen and are therefore not included in this subgroup analysis). These subgroups will be defined prior to breaking of the randomization code.

5. Graphical description of HbA1c over time, since the number of measurements during the study will vary and are not taken at fixed times, these measurements will be assigned to groups, for example 3,6,9,12 months +/- 1.5 month, potentially less groups in case of a lower number of measurements in order to ensure that all patients have a value at each curve point.

6. Secondary endpoints:

- Reported minor hypoglycaemia/major hypoglycaemia during the entire treatment period and in the last three months. Fishers Exact test, (moreover, in case of need for a more detailed analysis, that is significant effect on the primary endpoint and tendency to a difference of hypos) Cox regression with hypoglycaemia as recurring endpoint with treatment as covariate in a gamma frailty model).
- total end of treatment insulin dose, adjusted primarily for the baseline dose and baseline HbA1c thereafter also for weight and BMI.
- end of treatment body weight, BMI, waist-hip ratio adjusted for baseline values and with treatment as fixed effect.
- end of treatment blood pressure, lipids, geometic mean of urinary albumin excretion at end of treatment, analysed in ANOVA adjusted for baseline with treatment as fixed effect and demographic variables as covariate.
- For secondary endpoints, except for adverse events, there will be made last observation carry forward as with the analysis for the primary endpoint (in practice, this is only possible for the insulin dose – for other variables, and for patient number 10054 - see later note about this).
- All adverse events in the included patients and separately for the intention to treat population will be included in the analysis no matter the start time for the event during the study period.

7. In the protocol there are specified various secondary endpoints which can be determined by external laboratories (Coen Stehouwer) and analysed as previously described for HbA1c. Also, the association between changes in Hba1c and endothelial/coagulation/inflammatory markers and other factors which have changed significantly by the treatment can be evaluated with regression and potentially by GEE (generalized estimation equations) according to Coen Stehouwer.

8. Definition of the analysis population (modified according to the statistical analysis plan from the Reform-Study – paper on file):

The Intention To Treat (ITT) population will consist of all subjects who are randomised and exposed to at least one dose of trial medication. Trial medication will be interpreted as metformin or corresponding placebo tablets.

The Per Protocol (PP) population will consist of all subjects in the ITT population who completed the 13 month intervention period, except subjects who have violated the protocol in a manner likely to influence the primary endpoint. The following are possible reasons for excluding subjects from the PP population:

- violation of inclusion or exclusion criteria
- violation of protocol procedures, such as not following the treatment regimen

For the run-in period separate ITT and PP populations will be defined by a similar algorithm in order to evaluate drop-outs prior to randomisation.

The decision to exclude subjects from the ITT or PP population will be taken jointly by the medically responsible and the statistician. The decision will be taken before the breaking of the blind and it will be documented in a note describing the reason for exclusion of a subject.

Note on patient no. 10054:

This patient did not complete the 13 months study-period, but dropped out after 10 months. At 10 months the patient performed a full end-of-study visit (visit 3) including all secondary endpoints (in contrast to other drop-outs, who have missing values at end-of-study). The end-of-study visit samples (visit 3) for patient 10054 is labelled in the database as for end-of-study samples for patients with complete follow-up and will remain in intention-to-treat analyses as so (as last-obervation-carry-forward). No matter, patient 10054 is included in the drop-out population and not among study-completers. Therefore, patient no. 10054 must be excluded in analyses restricted to study-completers.
