# Supplementary material for: Effect of Adjunct Metformin Treatment in Patients with Type-1 Diabetes and Persistent Inadequate Glycaemic Control. A Randomized Study
Source: PLoS One. 2008 Oct 9;3(10):e3363. doi: 10.1371/journal.pone.0003363 (PMC2566605; doi:10.1371/journal.pone.0003363)
Supplement: Protocol S2 — Protocol and statistical analysis plan-Danish version. The study protocol and statistical analysis plan including all amendments prior to breaking of the blind. The original Danish version. (0.17 MB DOC) [file pone.0003363.s002.doc]

# Protokol ”Type-1-Metformin”

**Titel:**

**Effekten af metformin på glykæmisk kontrol og non-glykæmiske kardiovaskulære risikofaktorer hos patienter med type 1 diabetes, som vedvarende ikke er acceptabelt reguleret på behandling med insulin og diæt.**

**Kort titel:**

”Type-1-Metformin”

**Projektgruppe:**

Læge Søren Søgaard Lund; læge Peter Jacobsen; læge Minna Wittrup; læge, seniorforsker Lise Tarnow; ledende bioanalytiker Merete Frandsen; overlæge Hans-Henrik Parving; og overlæge Allan Vaag, Steno Diabetes Center.

**Undersøgelsesansvarlig:**

Allan Vaag og Hans-Henrik Parving.

**Sted:**

Steno Diabetes Center, Niels Steensens Vej 2, 2820 Gentofte, Danmark.

Tlf.: +45 39680800.

**Baggrund og rationale:**

Den øgede mortalitet og morbiditet hos patienter med diabetes skyldes overvejende udviklingen af senkomplikationer (1;2). Mere end 90% af patienter med type 1 diabetes vil med stigende diabetes-varighed udvikle varierende grader af diabetiske senkomplikationer (1). Forhøjet blodglucose er en vigtig faktor for udviklingen af alle typer senkomplikationer (1;2). En af de vigtigste måder at forebygge udvikling og hindre progression af senkomplikationer er ved forbedret regulering/normalisering af blodglucose-niveauet, dvs. forbedret glykæmisk kontrol (1;3). DCCT studiet er det største studie af effekten af forbedret glykæmisk kontrol hos patienter med type 1 diabetes. Studiet bestod af ca. 1500 patienter fulgt i 6.5 år (4) og viste, at man kunne reducere risikoen for senkomplikationer med 35-90% med intensiv blodglucose kontrol. Endvidere fandt man, at risikoen for senkomplikationer hang nøje sammen med den gennemsnitlige glykæmiske kontrol målt som HbA_1c_. En senere opfølgning af DCCT studiet har vist, at den gavnlige effekt af intensiv blodglucose-kontrol er vedvarende selv ved en beskeden ændring i gennemsnitligt blodglucose-niveau (0.4 % i HbA_1c_)(3). Et afgørende mål i behandlingen af patienter med type 1 diabetes er derfor at forbedre den glykæmiske kontrol uden betydende bivirkninger.

Hidtil har reguleringen af blodglucose-niveauet hos patienter med type 1 diabetes bestået af behandling med insulin og diæt. Denne behandling er ikke tilstrækkelig i daglig klinisk praksis til at opnå det anbefalede glykæmiske kontrol (HbA_1c_ < 7,5%) hos ca. 60-70% af patienterne på Steno Diabetes Center. De seneste data fra kvalitetssikrings-databasen på Steno Diabetes Center viser, at hos patienter med type 1 diabetes ligger ca. 10% med HbA1c højere end 12% (upublicerede data). Der eksisterer på nuværende tidspunkt ingen andre behandlingsprincipper end justering af insulin-dosis og diæt.

Metformin er et præparat som har været anvendt til behandling af type 2 diabetes gennem 30 år (1). Det virker ved bl.a. at øge kroppens følsomhed for insulin – insulin-sensitiviteten i musklerne og leveren samt sænke den hepatiske glucose-produktion (26). En vigtig del af årsagen til type 2 diabetes er nedsat følsomhed for insulin (insulin resistens). Metformin er første valgs præparat til overvægte patienter med type 2 diabetes (5). Behandling med metformin og nat-insulin hos overvægtige type 2 diabetikere er vist at være bedre til at sænke blodglucose-niveauet end insulin alene (6-9).

Mindre studier af patienter med type 1 diabetes uden senkomplikationer (10-13) og med begyndende diabetisk nyresygdom (14) tyder på at også disse patienter, ligesom patienter med type 2 diabetes, er insulin resistente.

Effekten af metformin hos patienter med type 1 diabetes er dårligt belyst. Et studie af 10 patienter med type 1 diabetes har vist at metformin øger insulin-sensitiviteten hos sådanne patienter (15) medens et netop publiceret studie af 27 teenagere med type 1 diabetes ikke kunne vise effekt af metformin på insulin-sensitiviteten (25). Flere mindre studier har vist, at metformin har en insulin besparende effekt dvs., at patienternes døgn-behov for insulin mindskes (16-19, 24-25).

To nylige studier af metformin-behandling til patienter med type-1-diabetes har vist modstridende resultater, hvad angår effekten på glykæmisk kontrol. Det første af disse studier undersøgte effekten af metformin-behandling til patienter med type-1-diabetes behandlet med insulin-pumpe. Der indgik 62 voksne patienter (alder 40 år i gennemsnit), som havde en gennemsnitlig HbA_1c_ ved baseline på 7,58% - dvs. acceptabel glykæmisk kontrol. Studiet fandt ingen signifikant effekt på HbA_1c_ af metformin-behandling efter 6 måneder og ingen forskel i antal hypoglykæmi-episoder (24). Det andet studie undersøgte 27 teenagere med med type-1-diabetes og dårlig metabolisk kontrol (HbA_1c_ ved baseline henholdsvis 9,3% og 8,6% i gennemsnit i metformin vs. placebo-gruppen) og fandt en signifkant absolut forskel på 0,6% i HbA_1c_ (metformin vs. placebo) efter 3 måneders behandling. I den metformin-behandlede gruppe fandt man øget tendens til milde hypoglykæmiske episoder. Alvorlige hypoglykæmi-episoder fandtes i 2 tilfæde i metformin-gruppen og 1 tilfælde i placebo-gruppen (25). Begge studier bekræftede som anført tidligere studiers fund vedr. den insulin-besparende effekt af metformin.

De hidtidige studier af metformin-behandling til patienter med type-1-diabetes har således på en række områder vist modstridende resultater og har ikke haft den nødvendige størrelse eller længde til at konkludere, om metformin har en vedvarende gavnlig effekt på den glykæmiske kontrol og dermed langtids-prognosen hos patienter med type 1 diabetes (9). Det er således aldrig undersøgt om behandling med metformin som supplement til insulin behandling hos patienter med type 1 diabetes med dårlig metabolisk regulation forbedrer den glykæmiske kontrol vedvarende, dvs. medfører fald i HbA_1c_ efter minimum 12 måneders behandling.

Ud over den potentielt gavnlige effekt af metformin på glykæmisk kontrol, er der også beskrevet en række andre positive effekter af metformin. UKPDS studiet viste, at overvægtige patienter med type 2 diabetes som blev behandlet med metformin havde signifikant lavere forekomst af apoplexi, diabetes relaterede events samt total dødelighed sammenlignet med andre behandlinger med samme blodglucose-reducerende virkning (20). Andre studier har vist, at metformin muligvis sænker lipid-niveauet i blodet (kolesterol og triglycerid) hos patienter med type 2 diabetes (21). Et suboptimalt designet, mindre studie har vist, at metformin sænker blodtrykket og urin-albumin-udskillelsen hos 51 patienter med type 2 diabetes med begyndende diabetisk nyresygdom (mikroalbuminuri) (22). Disse forhold er ikke undersøgt hos patienter med type 1 diabetes.

De få studier, der har anvendt metformin til patienter med type 1 diabetes fandt, at behandlingen var veltolereret (16;18;19, 24,25).

Hvis det viser sig, at metformin kan forbedre den glykæmiske kontrol og evt. positivt påvirke andre risikofaktorer, vil det være et gennembrud i behandlingen af patienter med type 1 diabetes med dårlig metabolisk kontrol.

**Formål:**

*At undersøge effekten af metformin på den glykæmiske kontrol og non-glykæmiske kardiovaskulære risikofaktorer hos patienter med type 1 diabetes med vedvarende dårlig glykæmisk kontrol..*

**Materiale:**

100 mandlige og kvindelige patienter med type 1 diabetes. Patienterne vil primært blive rekrutteret fra Steno Diabetes Center og herudover via kontakt til andre endokrinologiske afdelinger i hovedstadsområdet eller eventuelt ved annoncering.

**Inklusionskriterier:**

- Type-1-diabetes (WHO-kriterier) (23)
- Alder > 18 år.
- Debut-alder < 35 år.
- Diabetes varighed > 5 år
- Faste-C-peptid: koncentration < 0,3 pmol/l.
- HbA_1c_ > 8,5% gennem det sidste år.

**Baseline karakteristika:**

Rutine-bestemmelse af parametre i relation til diabeteskontrol og andre specifikke endepunkter (se senere), diabetiske senkomplikationer, ASAT, Faktor 2,7,10, se-creatinin, EKG, elektrolytter, Hgb, DNA m.h.p. senere farmakogenetisk subanalyse, se-B12-vitamin, erotrocytfolat, homocystein samt antropometriske og socioøkonomiske data (køn, alder, højde, vægt, fedtfordeling, race, ryge- og motionsvaner, social status etc.). Ved inklusionen tages endvidere øvrige parakliniske tests sv.t. in- og eksklusionskriterier. Der registreres forekomst af sendiabetiske komplikationer ud fra journaloplysninger og anamnese. Patientens mening om årsagen til den dårlige glykæmiske regulation (frygt for hypoglykæmi, dårlig diæt- og injektions-compliance etc.) vil blive registreret.

For kvinder registreres hvorvidt de er præ- eller postmenopausale. For præmenopausale kvinder sikres at de benytter adækvat antikonception (p-piller, spiral eller depot-gestagen-injektion). Præmenopausale kvinder vil få lavet en graviditets-test (urin-HCG).

**Eksklusionskriterier:**

- Nyresygdom (s-creatinin over øvre normalgrænse for det pågældende køn).
- Leversygdom (ASAT forhøjet 3 gange over øvre normalgrænse, basisk phosphatase 2 gange over øvre normalgrænse eller Faktor 2-7-10 nedsat under nedre normalgrænse).
- Tegn til inkompenseret hjertesygdom.
- Alvorlige organiske eller metaboliske sygdomme inkl. cancer.
- Graviditet eller planlagt graviditet.
- Alkohol-misbrug eller andet misbrug (euforiserende stoffer).
- Ophævede varslings-symptomer ved hypoglykæmi.

**Studieprotokol- og design:**

Studiet er designet som et single-center, randomiseret, dobbelt-blindt, placebo-kontrolleret parallelstudie af metformin som supplerende antiglykæmisk behandling til igangværende insulin-behandling over en periode på 12 måneder. Observationsperioden på 12 måneder er valgt for at opnå stabil metabolisk regulation idet andre studier med kombination af metformin og insulin hos patienter med type 2 diabetes har vist, at HbA1c først stabiliseres efter 3-6 måneder (8).

Patienterne randomiseres til behandling med tablet metformin 500 mg, som optitreres til 1g x 2 dagligt til måltiderne eller tilsvarende placebo i tillæg til patientens igangværende insulin-behandling (interventions-perioden). Optitreringen af forsøgsmedicinen foretages med minimum 1 uges mellemrum. Start-dosis af metformin eller tilsvarende placebo er 500 mg x 1 stigende med 500 mg pr. uge til maximalt 1g x 2. Såfremt der opstår bivirkninger, der vurderes at være relateret til forsøgsmedicinen vil dosis heraf blive reduceret. Dette gælder såvel aktiv- som placebo-behandling idet behandlerne er blindet, hvorfor justering af forsøgsmedicin-dosis må foretages under antagelse af, at alle patienter får aktiv behandling.

Halvdelen af patienterne randomiseres til aktiv behandling og halvdelen til placebo. Før opstart af interventions-perioden vil der være en run-in-periode på 1 måned, hvor alle patienter behandles med placebo i enkeltblindt design. Dvs. at patienterne orienteres om, at de fra start i run-in-perioden behandles med enten metformin eller placebo. Alle patienter vil imidlertid blive behandlet med placebo i hele run-in-perioden (enkeltblindt). Ved baseline opstarter patienterne herefter interventions-perioden med metformin eller fortsat behandling med placebo. Patienterne bliver dog randomiseret (dobbeltblindt) til den efterfølgende behandlings-type (metformin eller placebo) i interventions-perioden allerede fra start i run-in-perioden. Herved opnås at tidspunktet for opstart af metformin-behandling maskeres for patienten således, at placebo-effekten i både run-in-perioden og interventions-perioden bliver maksimal. Behandlerne er ikke blindet i run-in-perioden. Den samlede varighed for hver patient er 13 måneder.

Der vil blive benyttet central randomisering samt blokrandomisering med uens blok-størrelse og frekvens. Behandlerne vil ikke være bekendt med randomiserings-blokkenes størrelse. Der vil herudover være to-trins kryptering af randomiserings-koden med behandlingerne kodet i 1. trin til henholdsvis behandling A eller B og 2. trin til metformin eller placebo. Patienterne stratificeres inden for de to behandlings-grupper efter HbA_1c_ og BMI, mhp. at opnå ens fordeling af disse parametre mellem de to behandlings-grupper.

Patienten vil inden opstart af forsøgsmedicinen modtage nøjagtig instruktion i indtag af tabletter ved projekt-sygeplejersken og optitreringen af dosis vil foregå ved telefonisk kontakt til projekt-sygeplejersken. Såfremt patienterne ikke selv er i stand til at monitorere blodglukose, vil de yderligere modtage en grundig instruktion i dette. Planen er, at patienterne i run-in-perioden og den sidste måned af undersøgelsen hjemmemonitorer 7 punkts-profiler henholdvis før og halvanden time efter hvert hovedmåltid samt inden sengetid to dage pr. uge. Patientens blodsukker-apparat vil blive kalibreret inden opstart i forsøget. Patienter, der ikke selv har et blodsukker-apparat i forvejen vil blive tilbudt at låne et apparat af Steno Diabetes Center.

Patienterne vil blive opfordret til i videst muligt omfang at fortsætte deres vanlige insulin-regime såvel, hvad angår dosis, tidspunkt, blodsukkermåling (udover de sidste 2 uger af henholdsvis run-in-periode og ved forsøgsperiodens afslutning) og injektions-teknik.

Såfremt, der opstår insulin-tilfælde, insulin-chok eller patienterne oplever en uacceptabel stigning i antallet af hypoglykæmier (f. eks. natlig hypoglykæmi) vil der udfra en samlet klinisk vurdering blive foretaget justeringer i insulin-dosis. I disse tilfælde vil insulindosis blive justeret individuelt i samråd mellem de fra studiet ansvarlige læger og sygeplejersker efter de gældende afdelings-instrukser på Steno Diabetes Center.

Andre behandlingsmodaliteter, udover antiglykæmisk medicin, vil blive fortsat eller opstartet i overensstemmelse med god klinisk praksis i undersøgelsesperioden. Art og dosis af denne medicin vil blive registreret ved hvert kontrolbesøg under forsøget. Det tilstræbes, at denne medicin holdes uændret under forsøget således, at ikke-vitale behov for justering i medicinen udskydes til efter forsøgets afslutning.

Alle patienter vil blive opfordret til at opretholde deres vanlige livsstil hvad angår kost- og motionsvaner i hele forsøgsperioden.

**Monitorering:**

Studiets hoved-endepunkter vil blive monitoreret umiddelbart før interventions-perioden (baseline) samt efter 12 måneder.

Herudover vil patienterne i løbet af forsøget blive set af læge med 3-4 måneders mellemrum. Patienterne ses af de til forsøget tilknyttede læger og sygeplejersker ved henholdvis inklusions- og baseline-besøg (1. og 2. besøg) samt ved besøget ved forsøgets afslutning. Ved hvert af disse besøg vil HbA_1C_, blodglukose, vægt, BT og døgn for urin-albumin x 3 blive monitoreret (samt øvrige endepunkt-parametre – se senere). Med hensyn til HbA_1C_ vil denne blive dobbelt-bestemt (dvs. to analyser fra 2 forskellige blodprøver taget samme dag) ved inklusion, baseline samt ved undersøgelsens afslutning. Ved de øvrige besøg vil HbA1c blive monitoreret med enkelt bestemmelse dvs. i alt 9 gange pr. patient.

Ved besøgene henholdsvis 3, 6 og 9 måneder efter randomisering ses patienterne af deres sædvanlige læge på Steno Diabetes Center i rutine-ambulatoriet på Steno Diabetes Center. Disse besøg foregår derfor efter sædvanlig rutine både hvad angår konsultationen og monitorering af parametre i relation til diabetes-kontrol (blodglukose, HbA_1c_, vægt, BT, albuminuri, lipider, øjenkontrol etc.). Ved problemer relateret til projekt-medicinen kan såvel patienten som øvrige behandlere på Steno Diabetes Center på ethvert tidspunkt rette henvendelse til de læger og sygeplejersker, der er tilknyttet forsøget, som herefter vil tage videre aktion. Patienterne vil derfor blive udstyret med et telefon-nummer til projekt-sygeplejersken. Patienterne skal under alle omstændigheder holde telefonisk kontakt med projekt-sygeplejersken indtil maximal bivirkningsfri dosis af projektmedicinen er nået samt i ugerne inden afslutning af forsøget mhp. blodsukker-målinger.

Patient-compliance vil blive vurderet ved tælling af tabletter. Patienter med oplagte compliance-problemer vil blive tilbudt brug af doseringsæske.

**Endepunkter:**

*Primært:*

- Ændring i HbA_1C_.

*Sekundære:*

- Absolut HbA_1c_.
- Antallet af dokumenterede plasma-glukosekoncentrationer < 2,5 mmol/l i hele undersøgelsesperiodens samt i løbet af de 3 sidste undersøgelses-måneder.
- Rapporterede insulinfølinger svære insulintilfælde under hele undersøgelsen og i de sidste 3 måneder.
- Total insulindosis

*Følgende sekundære endepunkter monitoreres ved baseline og ved sidste besøg efter 12 måneder:*

- Plasma-PAI-antigen, PAI-aktivitet, t-PA-antigen, t-PA aktivitet, trombocyt-aggregation, trombocyt-antal og serotonin-release fra trombocytter efter 10 min. hvile.

- Plasma-fibrinogen
- Serum-albumin
- Markører for endotel-funktionen inkl. Von Willebrand faktor, ICAM, VCAM, selectin og endothelin.
- Plasma-Homocystein.
- Asymmetrisk DiMethylArginine - ADMA.
- Urin albumin ekskretion målt i 3 døgn-urin-opsamlinger.
- Ambulant blodtryks-monitorering siddende efter minimum 10 minutters hvile.
- Fastende lipid-status (total-cholesterol, LDL-cholesterol, HDL-cholesterol, VLDL-cholesterol og triglycerider).
- Vægt, BMI, hofte-talje-ratio.
- Leucocytantal, CRP, IL-6 og TNF-alfa.
- Se-kreatinin, elektrolytter, ASAT, basisk phosphatase, faktor 2-7-10, B-12 vitamin, erytrocyt-folat samt Hgb-koncentrationen.

For alle analyser vil tidspunkt for henholdsvis prøvetagning og biokemisk analyse blive registreret.

Der vil blive taget ekstra blodprøveglas til nedfrysning, såfremt der senere bliver behov for yderligere analyser ud over ovennævnte.

**Varighed:**

Undersøgelsen vil blive påbegyndt ca. pr. 01.10.03, og inkludering af patienterne til undersøgelsen forventes afsluttet senest 01.04.05. Hermed forventes hele undersøgelsen at være afsluttet ca. 01.04.06 – dvs i alt 30 måneder.

**Anvendelighed af undersøgelsesresultaterne:**

Det forventes, at undersøgelsesresultaterne kan få afgørende betydning for behandlingen af patienter med type-1-diabetes med dårlig metabolisk regulation. Ligeledes vil en positiv effekt på non-glykæmiske kardiovaskulære risikofaktorer herunder urin-albumin-udskillelsen kunne formindske risikoen for udviklingen af sen-diabetiske komplikationer specielt hjerte-kar-sygdomme og diabetisk nyresygdom.

**Data:**

Kilde-data er ikke tidligere nedskrevet eller elektronisk registrerede data (registrering af sendiabetiske komplikationer vil dog foregå delvist ud fra tidligere journaloplysninger).

Kilde-data registreres i forsøgsrapporten såvel i elektronisk form som i nedskreven form. Kilde-data og randomiserings-kode opbevares hos Hans-Henrik Parving/Allan Vaag og Søren Lund på Steno Diabetes Center. Datatilsynets standardvilkår vedrørende sikkerhed vil blive fulgt og personidentificerbare oplysninger vil blive anonymiseret ved forsøgets afslutning.

Randomiseringskoden vil kun blive brudt såfremt dette er af vital betydning for en patients helbred eller funktionsevne. Kun den forsøgsansvarlige kan træffe beslutning herom. Der vil ikke blive indsamlet data efter ophør med forsøgsmedicinen.

Der etableres en biobank med nedfrosne prøver (plasma, serum, urin samt DNA-materiale) mhp. senere analyse. Disse prøver opbevares på ubestemt tid. Alle øvrige prøver destrueres umiddelbart efter analyse og senest ved projektets afslutning.

**Sikkerhedsvurdering:**

Patienterne vil, ved de ambulante kontroller, blive spurgt om eventuelle komplikationer under forsøget, og vil tillige blive opfordret til telefonisk at rapportere disse til undersøgerne i de mellemliggende perioder.

Der skelnes mellem tre typer komplikationer:

*Bivirkning:* Alle skadelige og utilsigtede reaktioner af forsøgs-medicinen uanset dosis.

*Hændelse:* Ethvert uønsket medicinsk tilfælde, der tilstøder en patient i forsøget og som ikke nødvendigvis har kausal sammenhæng med forsøgs-medicinen.

*Alvorlig bivirkning eller alvorlig hændelse:* Ethvert medicinisk tilfælde uanset dosis, som resulterer i død, er livstruende, medfører hospitalisering eller forlængelse af eksisterende hospitalisering, resulterer i vedvarende eller betydelig invaliditet/uarbejdsdygtighed eller er en medfødt anomali/misdannelse.

Alle bivirkninger og hændelser registreres med art, tidspunkt, varighed samt evt. følgevirkninger heraf eller behandling og rapporteres ved forsøgets afslutning til sponsorerne samt til Lægemiddelstyrelsen.

Alle alvorlige bivirkninger eller alvorlige hændelser vil omgående blive rapporteret til både sponsorer og Lægemiddelstyrelsen.

Der vil ikke blive registreret evt. komplikationer efter forsøgets afslutning.

Evt. komplikationer i forsøgsperioden vil blive fulgt op og søgt behandlet i overensstemmelse med god klinisk praksis . Monitoreringen af endepunkts- og baseline-parametrene vil medvirke til at imødegå, at der opstår komplikationer.

Estimeret blodtab ved undersøgelserne på baseline og efter 12 måneder: ca. 100 ml. ved hver af de to undersøgelser. Ved de øvrige kontroller er blodtabet svarende til et almindeligt rutine-besøg i diabetes-ambulatorium eller hos egen læge.

**Statistiske overvejelser og styrke-beregning:**

Patienter der behandles med placebo vil blive sammenlignet med patienter, der behandles med metformin. Resultaterne udtrykkes i mean og SD, hvis data er normalt fordelt, ellers som median (IQR). Der vil for alle variable blive udregnet ændringer gennem forsøget samt absolutte værdier. For Hba_1c_ vil alle målte værdier under behandling indgå i vurderingen af behandlingseffekten. Andre normal fordelte variable vil blive sammenlignet med uparret t-test. Ikke normal fordelte variable sammenlignes med non-parametriske test.

*Styrkeberegning*
For at beskrive det nødvendige antal patienter har vi anvendt variationen (SD=1,0%) på HbA_1c_ hos 240 type 1 patienter mindre end 42 år med HbA_1c_ > 9,0% fra Steno Diabetes Center.

Styrkeberegningen viste, at mindst 45 patienter kræves i hver gruppe for at påvise en absolut forskel i HbA_1c_ på 0,6% mellem de to arme, med alpha = 5% (to-sidet) og beta = 20%.

Med et forventet drop-out på ca. 10% estimeres det derfor, at der i alt skal randomiseres 100 patienter. Eventuelle drop-outs efter randomisering vil ikke blive erstattet.

**Etiske overvejelser:**

Studiet vil blive udført i henhold til principperne anbefalet i Helsinki Deklarationen efter godkendelse af den regionale etiske komité.

Al deltagelse i forsøget er frivillig. Tilsagn om deltagelse kan på et hvilket som helst tidspunkt før eller under forsøget trækkes tilbage af patienten.

Patienterne vil modtage mundtlig og skriftlig information om forsøget inden afgivelse af samtykke. Indholdet i den mundtlige information vil svare til indholdet i den skriftlige information og vil blive givet på et tidspunkt, der aftales forud med patienten. Den mundtlige information gives i uforstyrrede fysiske rammer af en af de til forsøget tilknyttede læger. Den mundtlige information gives efter den skriftlige information og inden inklusion i forsøget. Den skriftlige information sendes pr. brev til patienten forud for samtalen. Patienten vil blive tilbudt at medbringe en bisidder til samtalen samt tilbydes betænkningstid af minimum 1 døgns længde mellem den mundtlige information og afgivelse af skriftligt samtykke.

Udover den viden forsøgets resultater vil give om den optimale behandling af type 1 diabetes, vil der ikke være forbundet umiddelbare fordele for patienterne ved at deltage i forsøget.

Al medicin vil blive givet gratis til patienterne i undersøgelsesperioden. Der vil ikke blive givet honorar for deltagelse til patienterne.

Ubehaget ved deltagelse vil primært være bivirkningerne fra metformin og insulin behandling dvs. gastrointestinale symptomer, hypoglykæmi, udslet, hovedpine og træthed. Herudover tidsforbruget og den aktive deltagelse til kontroller, faste, døgnurin-opsamling, blodtryksmåling, hjemmeblodglukose monitorering samt ubehaget ved blodprøvetagning.

Behandlerne i undersøgelses-gruppen er alle ansat på Steno Diabetes Center, der ejes og delvis administreres af Novo Nordisk A/S. Behandlerne vil ikke personligt opnå nogen økonomisk eller profesionelle fordele før, under eller efter forsøget.

Særligt bemyndigede personer fra Etisk Komitè eller Lægemiddelstyrelsen vil under hele forsøget have uhindret adgang til monitorering, auditering og inspektion af kilde-data/dokumenter vedrørende forsøgets patienter, herunder disses patient-journaler fra Steno Diabetes Center.

Novo Nordisk A/S kan tilbageholde data i indtil 4 måneder, men kan herefter ikke tilbageholde data fra publikation.

**Forsikringsforhold:**

Patienterne er på sædvanlige vilkår dækket af produkt-ansvars-forsikringen for forsøgsmedicinen samt Patientforsikrings-loven.

**Publicering og anmeldelse:**

Alle forsøgets resultater vil blive publiceret i et internationalt tidsskrift. Det praktiske arbejde i forsøget samt sammenskrivning af data vil overvejende blive foretaget af Søren Søgaard Lund med hjælp fra de øvrige behandlere i undersøgelsesgruppen. Forsøget vil blive anmeldt til Datatilsynet og Lægemiddelstyrelsen.

**Gennemførlighed af projektet:**

Alle de i undersøgelsen indgående endepunkter er implementeret og benyttes dagligt på Steno Diabetes Center af de i undersøgelsen indgående læger. De givne behandlingsregimer benyttes rutinemæssigt i den daglige kliniske praksis hos andre patient-grupper på Steno Diabetes Center. Protokollen vil blive forelagt hospitalsledelsen på Steno Diabetes Center m.h.p. afklaring og fuld dækning af ressourceforbruget.

**Budget:**

Undersøgelses-læge 30 mdr: ca. 1.000.000 kr.

½ Laborant 30 mdr.: ca. 500.000 kr.

½ Sygeplejerske 30 mdr.: ca. 500.000 kr.

Laboratorieanalyser m.m.: ca. 200.000 kr.

Transport af patienter: ca. 100.000 kr.

Pakning og mærkning af tabletter via KLIFO: ca. 50.000 kr.

**TOTAL:**   **2.350.000 kr.**

Herudover vil der være udgifter til forsøgsmedicinen i 12 måneder:

*Metformin-tabletter:* 4 piller dagligt pr. patient x (366 dage + ekstra 31 dage) = 1.588 tabletter pr. patient = 16 beholdere á 100 stk. tabletter pr. patient = 1600 x (50 patienter + ekstra 5 patienter) *= 88.000 tabletter* (samme mængde placebo tabletter skal bruges = *88.000 tabletter* Metformin-placebo-tabletter). Til run-in perioden regnes med et placebo-tablet forbrug på: 1 tablet dagligt pr. patient x (31 dage + ekstra 31 dage) = 62 tabletter pr. patient = 1 beholder á 100 stk. pr. patient = 100 tabletter x (100 patienter + 50 ekstra patienter) = *15.000 tabletter.*

Total antal Metformin-placebo-tabletter: 15.000 + 88.000 = *103.000 tabletter*

I ovenstående regnestykke indgår ekstra 31 dages behandling i både run-in-periode og interventions-periode for at gøre møde-datoerne mere fleksible for patienter og behandlere. Ligeledes regnes med ekstra 50 patienter til run-in-perioden idet det forventes, at en del patienter udgår i run-in-perioden bl. a. pga. dårlig compliance. Da alle patienter randomiseres til et medicin-nummer fra starten af run-in-perioden vil det ekstra antal placebo-tabletter muliggøre, at et medicin-nummer kan genanvendes fra de patienter der er udgået i run-in-perioden. Herved undgår man at patienter, der udgår i run-in-perioden optager et medicin-nummer til interventions-perioden. I interventions-perioden regnes med medicin til 10 ekstra (5 ekstra metformin + 5 ekstra metformin-placebo) randomiserede patienter. Denne medicin skal bruges i tilfælde af, at enkelte patienter f. eks. mister større mængder af forsøgsmedicinen eller ikke kan møde til kontrol inden for de givne tidsfrister samt til kontrol af pakke-proceduren hos KLIFO.

Udgifterne til henholdsvis læge-løn i 30 måneder (kr. 1.000.000,-) og ½ sygeplejerske i 30 måneder (kr. 500.000,-) er dækket via budgettet i andre igangværende studier på Steno Diabetes Center. Diabetesforeningen har endvidere bevilget støtte på kr. 100.000,- under forudsætning af projektets godkendelse fra etisk komité. Den resterende del som endnu ikke er dækket udgør derfor: kr. 2.350.000 – 1.500.000 – 100.000 = **750.000,- kr.**

GEA A/S har bevilget støtte til projektet i form af levering af metformin-tabletter og tilsvarende placebo.

Patienter i insulin-behandling er berettiget til gratis test-materialer jf. Lov om social service §97.

Der vil til undersøgelsen blive ansøgt om støtte fra kliniske udviklingspuljer på Steno Diabetes Center, fra alle relevante offentlige fonde inkl. Diabetesforeningen samt fra medicinalindustrien.

**Referencer:**

(1) Medicinsk Kompendium. 15 udgave ed. Nyt Nordisk Forlag, Arnold Busck, 2000.

(2) Parving H-H, Østerby R, Ritz E. Diabetic nephropathy. In: Brenner BM, Levine S, editors. The Kidney. Philadelphia: WB Saunders, 2000: 1731-1773.

(3) Effect of intensive therapy on the microvascular complications of type 1 diabetes mellitus. JAMA 2002; 287(19):2563-2569.

(4) The effect of intensive treatment of diabetes on the development and progression of long-term complications in insulin-dependent diabetes mellitus. The Diabetes Control and Complications Trial Research Group. N Engl J Med 1993; 329(14):977-986.

(5) Type 2-diabetes og det metaboliske syndrom - diagnostik og behandling. Ugeskr Læger 2000; Klaringsrapport nr. 6.

(6) Giugliano D, Quatraro A, Consoli G, Minei A, Ceriello A, De Rosa N et al. Metformin for obese, insulin-treated diabetic patients: improvement in glycaemic control and reduction of metabolic risk factors. Eur J Clin Pharmacol 1993; 44(2):107-112.

(7) Robinson AC, Burke J, Robinson S, Johnston DG, Elkeles RS. The effects of metformin on glycemic control and serum lipids in insulin-treated NIDDM patients with suboptimal metabolic control. Diabetes Care 1998; 21(5):701-705.

(8) Yki-Jarvinen H, Ryysy L, Nikkila K, Tulokas T, Vanamo R, Heikkila M. Comparison of bedtime insulin regimens in patients with type 2 diabetes mellitus. A randomized, controlled trial. Ann Intern Med 1999; 130(5):389-396.

(9) Daniel JR, Hagmeyer KO. Metformin and insulin: is there a role for combination therapy? Ann Pharmacother 1997; 31(4):474-480.

(10) Lager I, Lonnroth P, von Schenck H, Smith U. Reversal of insulin resistance in type I diabetes after treatment with continuous subcutaneous insulin infusion. Br Med J (Clin Res Ed) 1983; 287(6406):1661-1664.

(11) Lonnroth P, Blohme G, Lager I, Tisell LE, Smith U. Insulin resistance in fat cells from insulin-treated type I diabetic individuals. Diabetes Care 1983; 6(6):586-590.

(12) Yki-Jarvinen H, Koivisto VA. Insulin resistance in type I diabetes: prevalence, pathogenesis and therapeutic approaches. Ann Clin Res 1984; 16(2):74-83.

(13) Ekstrand AV, Groop PH, Gronhagen-Riska C. Insulin resistance precedes microalbuminuria in patients with insulin- dependent diabetes mellitus. Nephrol Dial Transplant 1998; 13(12):3079-3083.

(14) Yip J, Mattock MB, Morocutti A, Sethi M, Trevisan R, Viberti G. Insulin resistance in insulin-dependent diabetic patients with microalbuminuria. Lancet 1993; 342(8876):883-887.

(15) Gin H, Messerchmitt C, Brottier E, Aubertin J. Metformin improved insulin resistance in type I, insulin-dependent, diabetic patients. Metabolism 1985; 34(10):923-925.

(16) Gin H, Slama G, Weissbrodt P, Poynard T, Vexiau P, Klein JC et al. Metformin reduces post-prandial insulin needs in type I (insulin- dependent) diabetic patients: assessment by the artificial pancreas. Diabetologia 1982; 23(1):34-36.

(17) Janssen M, Rillaerts E, De L, I. Effects of metformin on haemorheology, lipid parameters and insulin resistance in insulin-dependent diabetic patients (IDDM). Biomed Pharmacother 1991; 45(8):363-367.

(18) Pagano G, Tagliaferro V, Carta Q, Caselle MT, Bozzo C, Vitelli F et al. Metformin reduces insulin requirement in Type 1 (insulin-dependent) diabetes. Diabetologia 1983; 24(5):351-354.

(19) Schatz H, Winkler G, Jonatha EM, Pfeiffer EF. Studies on juvenile--type diabetes in children. Assessment of control under treatment with constant and variable doses of insulin with or without addition of biguanides. Diabete Metab 1975; 1(4):211-220.

(20) Effect of intensive blood-glucose control with metformin on complications in overweight patients with type 2 diabetes (UKPDS 34). UK Prospective Diabetes Study (UKPDS) Group. Lancet 1998; 352(9131):854-865.

(21) Wulffele MG, Kooy A, De Zeeuw D, Stehouwer CD, Gansevoort RT. The effect of metformin on blood pressure, plasma cholesterol and triglycerides in type 2 diabetes mellitus; a systematic review. Br J Clin Pharmacol 2002; 53(5):549P-550P.

(22) Amador-Licona N, Guizar-Mendoza J, Vargas E, Sanchez-Camargo G, Zamora-Mata L. The short-term effect of a switch from glibenclamide to metformin on blood pressure and microalbuminuria in patients with type 2 diabetes mellitus. Arch Med Res 2000; 31(6):571-575.

(23) WHO Report Part 1: Diagnosis and Classification of Diabetes Mellitus 1999.

(24) Meyer L et al: The Benefits of Metformin Therapy During Continuous Subcutaneos Insulin Infusion Treatment of Type 1 Diabetic Patients. Diabetes Care 25: 2153-2158, 2002.

(25) Hamilton J et al: Metformin as an Adjunct Therapy in Adolescents With Type 1 Diabetes and Insulin Resistance. Diabetes Care 26: 138-143, 2003

(26) Dunn J and Peters D.H.: Metformin. Drugs 1995, 49 (5): 725-729.

Dato: 27.06.2003.

Internt amendment til protokollen: ”Effekten af metformin på glykæmisk kontrol og non-glykæmiske kardiovaskulære risikofaktorer hos patienter med type 1 diabetes, som vedvarende ikke er acceptabelt reguleret på behandling med insulin og diæt” Kort titel ”Type-1-Metformin”.

Numrene henfører til ICH Guideline i GCP.

Ad. 6.1.1:

Dato: 29/4 2003. Protokolkode: KA 03046gs.

Ad. 6.1.3:

Sponsor (levering af forsøgs-medicin):

GEA Ltd., Kanalholmen 8-12, 2650 Hvidovre, Danmark. Reference-person: Mette Elbæk.

Sponsor-Investigator:

Steno Diabetes Center

Niels Steensensvej 2

2820 Gentofte

Reference personer: Overlæge Allan Vaag og Overlæge Hans-Henrik Parving.

Ad 6.1.4

Undersøgelses-ansvarlig:

Overlæge Allan Vaag, tlf: +45 30 75 02 34.

Ansvarsfordeling for projekt-gruppens deltagere:

Overlægerne Allan Vaag og Hans-Henrik Parving:

Overordnet ansvarlig for projektets gennemførelse, patient-behandling og overholdelse af de etiske regler.

Lægerne Lise Tarnow, Minna Wittrup, Peter Jacobsen og Søren Søgaard Lund:

Ansvarlig for den praktiske afvikling af projektet herunder patient-rekruttering og -behandling, medicin-håndtering, data-indsamling og –analyse/-publikation.

Ledende Bioanalytiker Merete Frandsen:

Overordnet ansvarlig for de biokemiske analyser af blod-, urin- og vævsprøver.

Bioanalytikerne Ulla Meng Schmidt, Birgitte Vilsbøl Hansen, Berit Rud Hansen, Lotte Pietznak, Tina Ragholm Juul og Ingelise Rossing:

Ansvarlig for den umiddelbare prøvetagning og håndtering af blod-, urin- og vævs-prøver til biokemisk analyse.

Sygeplejerske Bente Blaaholm Nielsen:

I fælleskab med projektets læge-gruppe ansvarlig for den praktiske afvikling af studiet herunder patient-rekruttering og –behandling, samt medicin-håndtering.

Sekretær Ingelise Holstein:

Central randomisering – dvs. allokering af patienter til de to behandlings-grupper.

Ad 6.1.7: Se under pkt. 6.1.3.

Ad 6.1 (Resume):

Formål:

At undersøge effekten af metformin på den glykæmiske regulation samt øvrige kardiovaskulære risiko-faktorer hos patienter med type-1-diabetes, som vedvarende er dårligt reguleret på behandling med insulin og diæt.

Baggrund:

90% af patienter med type-1-diabetes vil udvikle sendiabetiske komplikationer i form af mikro- eller makrovaskulære sygdom samt neuropati. Dårlig glykæmisk kontrol er en vigtig risiko-faktor for udvikling af disse komplikationer. DCCT-studiet viste, at forbedret glykæmisk kontrol kan forebygge eller hindre progression i disse senkomplikationer. Hidtil har insulin-behandling og diæt været eneste mulighed for forbedring af den glykæmiske regulation hos patienter med type-1-diabetes. En stor del af disse patienter har dog trods intensiv insulin-behandling fortsat vedvarende dårlig glykæmisk kontrol.

Stoffet Metformin har hos patienter med Type-2-diabetes vist at kunne forbedre den glykæmiske kontrol i kombination med insulin samt mindske risikoen for udviklingen af makrovaskulære komplikationer. Kun få og små studier har undersøgt Meformin-behandling til patienter med type-1-diabetes. Disse studier har antydet en positiv effekt af Metformin til denne patient-kategori.

Metode:

100 patienter med type-1-diabetes ved vedvarende dårlig glykæmiske kontrol dvs. HbA1c > 8,5%. Alle patienter behandles først 1 måned med placebo. Heefter behandles halvdelen med metformin og den anden halvdel fortsætter med placebo gennem 12 måneder. Alle patienter fortsætter deres eksisterende insulin-behandling uændret. Før og efter opstart af supplerende Meformin-behandling vurderes dels glykæmisk kontrol samt effekten på en række kendte kardiovaskulære risiko-faktorer f. eks. blodtryk, lipid-status, urin-albumin-udskillelse m.m.

Anvendelse:

Undersøgelsen vil vise, om Metformin-behandling kan forbedre den glykæmiske regulation og dermed prognosen hos patienter med type-1-diabetes med vedvarende dårlig glykæmisk kontrol trods intensiv insulin-behandling. Denne patient-gruppe er, pga. den dårlig glykæmiske kontrol i særlig høj risiko for at udvikle invaliderende sygdomme i en række organsystemer og heraf nedsat livskvalitet og livslængde.

Ad 6.2.5:

Forsøget vil efterleve GCP-reglerne i videst muligt omfang. Da forsøget allerede er anmeldt og godkendt hos Datatilsynet, Etisk komité og Lægemiddelstyrelsen som et ikke-GCP monitoreret studie vil der blive udarbejdet supplerende interne arbejds-dokumenter, som anviser retnings-linjer for forsøgets udførelse i så god overensstemmelse med GCP-reglerne som muligt. Disse retnings-linjer vil være i overenstemmelse med såvel den allerede godkendte protokol som med GCP-reglerne. Dog vil patient-informationen blive forelagt Etisk komité til fornyet vurdering mhp. godkendelse af, at GCP-enheden ved Københavns Universitets-hospital bevilges mulighed for at monitorere person-henførbare oplysninger blandt forsøgets data.

Ad. 6.4.2:

Forsøgstype og design: Single-center, dobbeltblindt, randomiseret, placebo-kontrolleret, parallel-studie.

Ad 6.4.3:

Der vil blive benyttet central randomisering samt blokrandomisering med uens blok-størrelse og frekvens. Behandlerne vil ikke være bekendt med randomiserings-blokkenes størrelse. Der vil herudover være to-trins kryptering af randomiserings-koden med behandlingerne kodet i 1. trin til henholdsvis behandling A eller B og 2. trin til metformin eller placebo. Patienterne stratificeres inden for de to behandlings-grupper efter HbA_1c_ (større eller mindre end 9,5%) og BMI (større eller mindre end 25 kg/m^2^), mhp. at opnå ens fordeling af disse parametre mellem de to behandlings-grupper. Den centrale randomisering forestås på Steno Diabetes Center af en medarbejder uden anden tilknytning til forsøget. Allokeringen af patienter til de to behandlings-grupper foretages som anført ud fra fastsatte kriterier for henholdsvis HbA_1c_ og BMI.

Ad 6.4.6:

Patienten kan ikke fortsætte forsøget, såfremt der opstår alvorlige uventede hændelser (”hændelser” er defineret i protokollen side 9) relateret til forsøgs-medicinen. Undtaget herfor dog enhver form for hypoglykæmi, da dette ikke anses for uventet. Forsøget afbrydes, såfremt der fremkommer oplysninger som viser, at risikoen ved at gennemføre forsøget er for stor, eller hvis andre forsøg viser, at behandling med metformin som supplement til insulin er bedre end insulin-behandling alene.

Ad 6.5.2:

Ved ”inkompenseret hjerte” forstås at patienten klassificeres som havende hjerteinsufficiens NYHA klasse I-II med Ejection Fraction < 30%, NYHA klasse III-IV eller såfremt objektive fund tyder på hjerteinsufficiens dvs. ødemer med kendt kardiel genese eller stase/3. hjertelyd ved hjerte-lunge-stetoskopi.

Ved ”Alvorlige organiske eller metaboliske sygdomme inkl. cancer” menes sygdomme, der forventes, at kunne afkorte patientens forventede levetid eller forringe patientens velfærd i en grad der kan påvirke forløbet i forsøget eller, hvor eksperimental behandling findes kontraindiceret af etiske årsager f. eks. aktuel cancer-diagnose eller skizofreni. Patienter med en tidligere cancer-diagnose, hvor behandlingen skønnes at have været kurativ (dvs., at patientens forventede levetid ikke er forringet heraf) kan deltage i forsøget.

Ad 6.5.3:

Withdrawal-kriterier:

Disse vil være identiske med eksklusions-kriterierne jf. protokollen side 4.

Ad 6.5.3:

Data for udgåede forsøgspersoner:

Udgåede forsøgs-personer vil indgå i en intention-to-treat analyse.

Ad 6.6:

Vedr. medicin-nummer: Tillæg herom følger senere.

Ad 6.9.5:

Opgørelse af manglende data m.m. Tillæg herom følger senere.

Yderligere:

Hypoglykæmi med eller uden symptomer anses for en forventet bivirkning til behandlingen og vil derfor ikke blive indberettet som adverse event med mindre der er tale om behov for hospitals-indlæggelse. Hypoglykæmi vil blive registreret i CRF på linie med øvrige bivirkninger.

Læger, der kontrollerer patienten under forsøget vil alle være instrueret i at udspørge om adverse events, anden medicin og compliance med forsøgs-medicinen selv om det er patientens vanlige læge på Steno Diabetes Center.

Evt. udefra kommende læger vil alle få oprettet en journal på Steno Diabetes Center.

Der oprettes en DNA-biobank mhp farmakogenetisk analyse.

Annonce-forslag er endnu ikke udarbejdet og afventer stillingtagen fra projektgruppen til om patienter skal rekrutteres herved.

Der foreligger patient-information og informeret samtykke-erklæring fra etisk komite.

**Dato: 05.07.2004.**

DEN VIDENSKABSETISKE KOMITÉ

Stationsparken 27

2600 Glostrup

Tlf. 4322 2308 / 2309

Fax 4322 2288

**Anmeldelse til**

**Videnskabsetisk Komité**

**Tillægsprotokol/**

**Amendment/**

**Ændringsprotokol til: KA** 03046gs (her anføres komitéens registreringsnr. på den oprindelige protokol)

| Projekttitel (her anføres projekttitel på den oprindelige protokol):  **”Effekten af metformin på glykæmisk kontrol og non-glykæmiske kardiovaskulære risikofaktorer hos patienter med type 1 diabetes, som vedvarende ikke er acceptabelt reguleret på behandling med insulin og diæt”. Kort titel ”Type-1-Metformin”.** |
| --- |
| Hvori består ændringen/ændringerne: I **protokollen** ændres følgende:  Side 5 linie 10 fra neden: ”Planen er, at patienterne i run-in-perioden og den sidste måned af undersøgelsen hjemmemonitorer 7 punkts-profiler henholdvis før og halvanden time efter hvert hovedmåltid samt inden sengetid to dage pr. uge.” udgår.  Side 5 linie 2 fra neden: ” (udover de sidste 2 uger af henholdsvis run-in-periode og ved forsøgsperiodens afslutning)” udgår. |
| Med hvilken begrundelse ønskes ændringerne foretaget:  Forenkling og reduktion af patientens indsats som forsøgs-deltager. |

HUSK OGSÅ AT UDFYLDE BAGSIDEN - VEND

Dato: 24.08.2004

**Internt amendment nr. 2 til forsøget med titlen:**

”Effekten af metformin på glykæmisk kontrol og non-glykæmiske kardiovaskulære risikofaktorer hos patienter med type 1 diabetes, som vedvarende ikke er acceptabelt reguleret på behandling med insulin og diæt” Kort titel ”Type-1-Metformin”.

Ad ”Kort titel”:

Forsøgets korte titel ændres hermed i interne dokumenter til: ”Met-1”

Ad randomiserings-procedure:

Randomiserings-proceduren ændres hermed på følgende vis:

Patienterne gives ved inklusion i studiet (første besøg) et patient-nummer, som følger patienten gennem hele forsøget. Dette nummer noteres på den udleverede forsøgs-medicin til brug i run-in perioden (medicin-dumaer med rød etiket). Ved andet besøg (baseline) efter ca. 1 måned foretages randomisering efter tidligere beskrevet procedure. Stratificering foretages ud fra baseline-karakteristika efter nedenstående procedure. Ved randomiseringen gives patienten udover patient-nummeret et tre-cifret medicin-nummer. Dette medicin-nummer er fortrykt på alle randomiserede projekt-medicin dumaer (medicin-dumaer med hvid etiket), og der skal derfor ikke noteres yderligere på disse dumaer. Patienten gives derfor først randomiserings-nummeret efter run-in-perioden er gennemført i modsætning til, hvad der tidligere er beskrevet i protokollen, hvor patienten fra starten af run-in perioden fik tildelt randomiserings-nummeret. Som hidtil planlagt orienteres patienterne om, at de påbegynder forsøgs-medicinen fra starten af run-in perioden og herved maskeres fortsat tidspunktet for opstart af aktiv-/placebo-behandling.

Ad stratificering:

Stratificerings-proceduren foretages med fortløbende numre (dvs. en patient, som skal randomiseres gives næste ledige fortløbende medicin-nummer i henholdsvis stigende/faldende orden jf. nedenstående tabel) i 4 lige store grupper ud fra baseline-værdier af henholdsvis HbA1c og BMI. De 4 grupper er følgende:

1. HbA1c ≥ 9,6% og BMI ≥ 25 kg/m^2^ starter medicin-numrene 1-25 (start nedefra 1, 2, 3 osv.)
2. HbA1c ≥ 9,6% og BMI < 25 kg/m^2^ starter medicin-numrene 26-50 (start oppefra 50, 49, 48 osv.)
3. HbA1c < 9,6% og BMI ≥ 25 kg/m^2^ starter medicin-numrene 51-75 (start nedefra 51, 52, 53 osv.)
4. HbA1c < 9,6% og BMI < 25 kg/m^2^ starter medicin-numrene 100-76 (start oppefra 100, 99, 98 osv.)

Såfremt der ikke opnås en ligelig fordeling af patienter i de 4 grupper vil grupperne kunne slås sammen.

Primært kan gruppe 1 og 2 slås sammen og/eller gruppe 3 og 4 slås sammen afhængig af, hvilken af de 4 grupper, hvor alle medicin-numre er opbrugt. Såfremt to grupper slås sammen vil samme tildeling af fortløbende numre henholdvis stigende/faldende blive anvendt uændret hos en given patient-gruppe. Hvis f. eks. gruppe 1 og 2 slås sammen, fordi alle medicin-numre i gruppe 1 er brugt, vil næste patient, som skulle have været i gruppe 1, blive overført til gruppe 2, men vil fortsat blive givet et medicin-nummer startende nedefra dvs. fra nummer 26, 27, 28 osv. og tilsvarende ved andre gruppe-sammenlægninger.

Såfremt der bliver fyldt i en gruppe, som allerede er slået sammen (f. eks. gruppe 1 og 2 eller 3 og 4) og der fortsat er ledige medicin-numre i de to sidste grupper vil de to tilbageværende grupper blive slået sammen og herefter tildeles patienter et medicin-nummer udelukkende ud fra HbA1c-værdi uafhængigt af BMI. Dette skyldes, at det skønnes vigtigere at fordele HbA1c jævnt mellem de to behandlings-grupper end BMI. HbA1c-grænsen er fortsat <9,6%/≥9,6% og i den sammenlagte gruppe vil den af gruppens to tidligere BMI-grupper med hidtil flest randomiserede patienter fortsat blive tildelt fortløbende stigende eller faldende numre fra patienter tilhørende samme HbA1c-gruppe. Den anden HbA1c-gruppe starter så med at tildeles numre fra den modsatte ende af den sammenlagte gruppe. Hvis der er randomiseret lige mange patienter i hver gruppe tildeles patienten det laveste ledige medicin-nummer og patienter fra den modsatte HbA1c-gruppe starter/fortsætter så fra modsatte ende af den sammenlagte gruppe.

Hvis f.eks. gruppe 3 og 4 slås sammen og der hidtil er randomiseret 7 patienter i gruppe 3 og 15 patienter i gruppe 4 så skal patienter med HbA1c < 9,6% tildeles numre oppefra i gruppe 4 uafhængigt af BMI og patienter med HbA1c ≥ 9,6% tildeles numre nedefra i gruppe 3 også uafhængigt af BMI og så fremdeles. Hvis der derimod var randomiseret lige mange patienter i gruppe 3 og 4 så ville næste patient skulle tildeles numre fra gruppe 3 og patienter fra den modsatte HbA1c-gruppe tildeles numre fra gruppe 4.

Ad publicering:

Ved publikation af studiet i videnskabeligt tidsskrift eller lignende vil Søren Søgaard Lund være første-forfatter på alle publikationer, med mindre anden skriftlig aftale mellem de involverede forfattere foreligger herom.

Ad in-/eksklusions-kriterier:

I protokollen med tilhørende amendment og (CRF) er desværre opstået en print-fejl således, at steder hvor der skulle have stået tegnene ≤ eller ≥ i stedet står < respektive >. Sidstnævnte tegn skal altså i alle in- og eksklusion-kriteriterier erstattes så < erstattes af ≤ og > erstattes af ≥. Dette gælder dog ikke eksklusion-kriterierne vedr. creatinin (som derfor fortsat vil være > 110 mikromol/l for kvinder og > 130 mikromol/l for mænd), ASAT (som fortsat vil være > 150) og Faktor II, VII, X (som fortsat vil være < 0,7) som det allerede fremgår af CRF’en.

Dvs. at inklusions-kriteriet for f. eks. HbA1c er ≥ 8,5% gennem det sidste år og så fremdeles.

Ad kontrol-besøg:

Som anført i protokollen foregår kontrollen af patienter efter opstart af randomiseret medicin i rutine-ambulatoriet på Steno Diabetes Center efter vanlig procedure bortset fra det afsluttende besøg i forsøget, som foregår i forsknings-afdeling (520) på Steno Diabetes Center. Registrering af bivirkninger til forsøgs-medicinen og opfølgning heraf, hvad angår evt. justering af forsøgs-medicin og/eller insulin vil dog blive foretaget primært via telefonisk kontakt af de til forsøget tilknyttede læger/sygeplejersker. Der vil være mindst 3 planlagte telefon-konsultationer fordelt jævnt over forsøgs-perioden pr. patient. Ligeledes kan registrering af bivirkninger blive foretaget ved at patienterne udfylder og indsender et spørgeskema herom, som så vil blive vurderet af de til forsøget tilknyttede læger/sygeplejerske mhp. behov for supplerende opfølgning. Såfremt, der bliver behov for ekstra ambulant kontrol herudover vil dette kunne foranstaltes i rutine-ambulatoriet eller i forsknings-afdelingen på Steno Diabetes Center. Besøgene i rutine-ambulatoriet vil ikke blive registreret i CRF’en. Oplysningerne til CRF’en vil blive indsamlet via den telefoniske kontakt/evt. spørgeskema som beskrevet.

Ad monitorering af HbA1c:

I protokollen fremgår, at HbA1c monitoreres 9 gange pr. patient i løbet af forsøget. Da monitoreringen af HbA1c i perioden mellem randomisering og afslutning foretages ved kontrol besøgene i rutine-ambulatoriet vil antallet af HbA1c målinger komme til at afhænge af antallet af disse kontrol-besøg og vil derfor variere mellem patienterne. Det er ikke planen, at HbA1c-målingerne fra rutine-ambulatoriet skal indgå som effekt-mål undtagen hos patienter, der udgår i randomiserings-perioden. For disse patienter kan HbA1c-målinger fra rutine-ambulatoriet indgå med sidst målte værdi som effektmål.

Ad blodsukker-apparat:

I protokollen anføres, at patienternes blodsukker apparat vil blive kalibreret ved forsøgets start. Da systematiske hjemme-blodsukker-målinger er udgået som effekt-mål i forsøget jf. tidligere protokol-amendment, skønnes der ikke længere at være behov for dette. Patienter vil dog efter vanlig procedure på Steno Diabetes Center blive tilbudt kalibrering af apparatet ved evt. behov herfor.

Ad Compliance:

Efter run-in perioden vil patientens forbrug af tabletter blive registreret. Compliance med forsøgs-medicinen i denne periode indgår dog ikke som kriterium for fortsat deltagelse i forsøget herunder deltagelse i randomiserings-perioden.

Dato: 27.10.2004.

**Internt amendment nr. 3 til forsøget med titlen:**

”Effekten af metformin på glykæmisk kontrol og non-glykæmiske kardiovaskulære risikofaktorer hos patienter med type 1 diabetes, som vedvarende ikke er acceptabelt reguleret på behandling med insulin og diæt” Kort titel ”Type-1-Metformin”.

Ad fastende C-peptid:

Der vil ikke, som det ellers fremgår af protokollen, på alle patienter blive målt fastende c-peptid inden inklusion eller randomisering. I stedet anvendes ikke-fastende c-peptid taget på inklusions-besøget (besøg 1) med identiske grænser som for fastende c-peptid. Såfremt denne er ≥ 300 pmol/l vil der blive foretaget supplerende test af beta-celle-funktionen f. eks. fastende c-peptid eller glukagon-test inden patienten kan randomiseres. Kun såfremt disse prøver tyder på type 1 diabetes kan patienten randomiseres.

Søren Lund 27/10 04.

Allan Vaag 27/10 04.

Dato: 22.12.2004.

*4. Addendum 27.12.04*

**Protokol: Met-1**

**Sponsor-investigator / forsøgsansvarlig læge: Allan Vaag**

**Ad inklusionskriterier:**

HbA1c ≥ 8,5% det sidste år

– betyder, at gennemsnitsværdien af målte HbA1c værdier skal være ≥ 8,5%.

Desuden skal HbA1c målt ved besøg 1 være ≥ 8,5%

- og på besøg 2 være ≥ 8,0%.

Levertal:

Ved en fejltagelse er der ikke blevet målt basisk fosfatase på alle patienter til inklusionsbesøget – da denne værdi er af mindre klinisk betydning vil patienter hvor værdier ikke foreligger dog kunne forblive i undersøgelsen. Der gøres et notat i CRF med henblik på klinisk relevans af manglende værdier.

Der vil blive ændret i laboratorie bestillingsproceduren, så vi kan sikre os at basiske fosfataser fremover måles ved besøg 1 på alle patienter.

Faktor 2,7,10 er af mindre klinisk betydning for patientens eligibility til studiet og ikke klinisk betydningsfulde afvigelser eller manglende værdier vil ikke få betydning for patientens deltagelse i studiet. Der gøres et notat i CRF med henblik på klinisk relevans af abnorme eller manglende værdier

**Ad screeningsperiode:**

Denne skal være på mindst 25 dage og maximalt på 100 dage.

### Til vitterlighed for dette dokument:

## Sponsor-investigator / forsøgsansvarlig læge

## Navn: _____________ Signatur: _____________Dato: __________________

Dato: 22.12.2006:

MET-1 analyse plan

(jvf protokol, oplæg fra SQRL og inspireret af K Hermansen Diabetes Care 29:1269,2006.

Randomiseret studie af effekten af metformin som tillæg til vanlig insulin behandling hos pt med type 1DM, 100 randomiseret og fulgt 1 år. Primær analyse intention to treat ( sidste HbA1c værdi og insulin-dosis, dog mindst 3 mdr efter randomisering bruges som endepunkt (last value carried forward) hvis pt udgår inden 12 mdr. Hvis pt udgår inden da kan HbA1c ikke evalueres meningsfyldt). Per protokol analyse kan laves ”explorativt” ved drop out for at vurdere behandlingens potentiale, men er sekundær til intention to treat analysen.

1. demografisk beskrivelse af de to randomiserede grupper mht fordeling af alder, køn, DM varighed, HbA1c, BMI insulin dosis/kg vægt, retinopati og u-alb kategori. Drop outs.
2. Analyse af primært endepunkt, effekt af metformin på HbA1c i ANOVA justeret for baseline HbA1c (ændring i HbA1c fra baseline i de 2 grupper) med behandling som fixed effect. Hvis randomiseringen har medført forskelle i væsentlige parametre (spec BMI) under pkt 1 justeres for disse i analysen. Det vurderes individuelt for hver parameter om forskellen er betydende.
3. Er den primære analyse negativ begrænses antallet (og værdien) af sekundære analyser. Dog laves under alle omstændigheder analyse som i pkt 2 justeret for BMI, samt en analyse stratificeret på median HbA1c og BMI ><25
4. sekundære analyser i øvrigt:
   1. som pkt 2 og 3 justeret for insulin resistens: ins/kg, talje/hofte ratio, eGDR (jvf T Orchard) samt køn, alder og DM varighed, samt for ændring i insulin dosis fra start til slut.(evt faste BS, C peptid, evt stratificeret på GAD antistoffer hvis forhøjet BMI øger effekten for at udelukke type 2, især hvis c peptid ikke er sikker negativ)
   2. Subgruppe-analyse af patienter, som i løbet af forsøgs-perioden skifter insulin-regime f. eks. fra human insulin til analog insulin og/eller ændrer antal daglige injektioner, opstarter pumpe-behandling e. lign. væsentlige ændringer i behandlings-regimet (ændringer i insulin-dosis betragtes ikke som en væsentlig ændring i behandlings-regimet og indgår derfor ikke i denne subgruppe-analyse). Disse sub-grupper defineres før randomiserings-koden brydes.
5. Grafisk beskrives HbA1c over tid, da antallet af målinger under studiet vil variere og ikke er taget til faste tider grupperes værdierne i feks 3, 6, 9, 12 mdr +-1,5mdr evt færre grupper hvis der er færre målinger for at tilstræbe at alle pt har en værdi svarende til hvert kurvepunkt.
6. Sekundære endepunkter.
   1. Rapporterede insulinfølinger/ svære insulintilfælde under hele undersøgelsen og i de sidste 3 måneder. Fishers Exact test, (endvidere ved behov for nærmere analyse (dvs signifikant effekt på primært endepunkt og tendens til forskel i antal hypo) Cox regression med hypo som recidiverende endepunkt, med behandling som covariat vha en gamma frailty model.)
   2. Total slut insulindosis, justeret primært for baseline dosis og baseline hba1c, dernæst desuden for vægt, BMI
   3. Slut vægt/BMI/ T/H justeret for baseline værdi og m behandling som fixed effect.
   4. Slut Blodtryk, lipider , Geomtrisk mean af u-alb til slut, analyseret i ANOVA justeret for baseline m behandling som fixed effect og demografiske variable som covariate.
   5. For sekundære endepunkter, bortset fra bivirkninger, laves last-obervation-carry-forward tilsvarende analysen af det primære endepunkt (i praksis er dette dog kun muligt for insulin-dosis - for øvrige variable desuden hos patient 10054 – se note herom).
   6. Alle bivirkninger hos alle inkluderede patienter og separat for intention-to-treat-populationen inkluderes i analysen uanset tidspunkt for opståen i studie-perioden.
7. I protokollen er specificeret fsk sekundære endpunkter som kan bestemmes af Coen og analyseres som ovenfor beskrevet for HbA1c. Endvidere kan sammenhænge mellem ændringer i HbA1c eller endothel/ koag faktoerer/inflammations markører samt andre faktorer der måtte ændres signifikant af behandlingen vurderes ved regression og evt GEE (generaliserede estimation equations) jvf Coen
8. Definition af analyse-populationerne (modificeret jf. den statistiske analyse-plan fra Reform-studiet):

The Intention To Treat (ITT) population will consist of all subjects who are randomised and exposed to at least one dose of trial medication. Trial medication will be interpreted as metformin or corresponding placebo tablets.

The Per Protocol (PP) population will consist of all subjects in the ITT population who completed the 13 month intervention period, except subjects who have violated the protocol in a manner likely to influence the primary endpoint. The following are possible reasons for excluding subjects from the PP population:

- violation of inclusion or exclusion criteria
- violation of protocol procedures, such as not following the treatment regimen

For the run-in period separate ITT and PP populations will be defined by a similar algorithm in order to evaluate drop-outs prior to randomisation.

The decision to exclude subjects from the ITT or PP population will be taken jointly by the medically responsible and the statistician. The decision will be taken before the breaking of the blind and it will be documented in a note describing the reason for exclusion of a subject.

Note on patient no. 10054:

This patient did not complete the 13 months study-period, but dropped out after 10 months. At 10 months the patient performed a full end-of-study visit (visit 3) including all secondary endpoints (in contrast to other drop-outs, who have missing values at end-of-study). The end-of-study visit samples (visit 3) for patient 10054 is labelled in the database as for end-of-study samples for patients with complete follow-up and will remain in intention-to-treat analyses as so (as last-obervation-carry-forward). No matter, patient 10054 is included in the drop-out population and not among study-completers. Therefore, patient no. 10054 must be excluded in analyses restricted to study-completers.
