# Supplementary material for: Effect of Adjunct Metformin Treatment in Patients with Type-1 Diabetes and Persistent Inadequate Glycaemic Control. A Randomized Study
Source: PLoS One. 2008 Oct 9;3(10):e3363. doi: 10.1371/journal.pone.0003363 (PMC2566605; doi:10.1371/journal.pone.0003363)
Supplement: Checklist S1 — CONSORT Checklist (0.10 MB DOC) [file pone.0003363.s003.doc]

CONSORT Statement 2001 - Checklist  

Items to include when reporting a randomized trial 
PAPER SECTION And topic 	Item 	Descriptor 	Reported on Page # 	
TITLE & ABSTRACT 	1 	How participants were allocated to interventions (e.g., "random allocation", "randomized", or "randomly assigned"). 	Abstract.	
INTRODUCTION Background 	2 	Scientific background and explanation of rationale. 	Introduction.	
METHODS Participants 	3 	Eligibility criteria for participants and the settings and locations where the data were collected. 	Methods: Participants and Table 1.	
Interventions 	4 	Precise details of the interventions intended for each group and how and when they were actually administered. 	Methods: Interventions.	
Objectives 	5 	Specific objectives and hypotheses. 	Introduction and Methods: Objectives.	
Outcomes 	6 	Clearly defined primary and secondary outcome measures and, when applicable, any methods used to enhance the quality of measurements (e.g., multiple observations, training of assessors). 	Methods:
Outcomes.	
Sample size 	7 	How sample size was determined and, when applicable, explanation of any interim analyses and stopping rules. 	Methods:
Sample size.	
Randomization --Sequence generation 	8 	Method used to generate the random allocation sequence, including details of any restrictions (e.g., blocking, stratification) 	Methods: Randomization – Sequence generation.	
Randomization --Allocation concealment 	9 	Method used to implement the random allocation sequence (e.g., numbered containers or central telephone), clarifying whether the sequence was concealed until interventions were assigned. 	Methods: Randomization – Allocation concealment.	
Randomization --Implementation 	10 	Who generated the allocation sequence, who enrolled participants, and who assigned participants to their groups. 	Methods: Randomization - Implementation.	
Blinding (masking) 	11 	Whether or not participants, those administering the interventions, and those assessing the outcomes were blinded to group assignment. If done, how the success of blinding was evaluated. 	Methods:
Blinding.	
Statistical methods 	12 	Statistical methods used to compare groups for primary outcome(s); Methods for additional analyses, such as subgroup analyses and adjusted analyses. 	Methods:
Statistical methods.	
RESULTS Participant flow 	13 	Flow of participants through each stage (a diagram is strongly recommended). Specifically, for each group report the numbers of participants randomly assigned, receiving intended treatment, completing the study protocol, and analyzed for the primary outcome. Describe protocol deviations from study as planned, together with reasons. 	Methods: Protocol deviations (with relation to the follow-up of patients).
Results: Subjects; Figure 1. 	
Recruitment 	14 	Dates defining the periods of recruitment and follow-up. 	Results: Recruitment.	
Baseline data 	15 	Baseline demographic and clinical characteristics of each group. 	Results:
Subject characteristics; Table 2.	
Numbers analyzed 	16 	Number of participants (denominator) in each group included in each analysis and whether the analysis was by "intention-to-treat". State the results in absolute numbers when feasible (e.g., 10/20, not 50%). 	Results:
Numbers analyzed, primary, secondary and ancillary outcomes, respectively.	
Outcomes and estimation 	17 	For each primary and secondary outcome, a summary of results for each group, and the estimated effect size and its precision (e.g., 95% confidence interval). 	Results: Primary, secondary outcomes; Table 3.	
Ancillary analyses 	18 	Address multiplicity by reporting any other analyses performed, including subgroup analyses and adjusted analyses, indicating those pre-specified and those exploratory. 	Results: Primary outcomes, secondary analyses; Ancillary analyses; Tables 4, 5 and 6.	
Adverse events 	19 	All important adverse events or side effects in each intervention group. 	Results: Adverse events and safety variables; Tables 4 and 5.	
DISCUSSION Interpretation 	20 	Interpretation of the results, taking into account study hypotheses, sources of potential bias or imprecision and the dangers associated with multiplicity of analyses and outcomes. 	Discussion: Primary, secondary and ancillary outcomes; Limitations	
Generalizability 	21 	Generalizability (external validity) of the trial findings. 	Discussion: Limitations.	
Overall evidence 	22 	General interpretation of the results in the context of current evidence. 	Discussion:
Conclusions.	

www.consort-statement.org 
