# Supplementary material for: Effect of Adjunct Metformin Treatment in Patients with Type-1 Diabetes and Persistent Inadequate Glycaemic Control. A Randomized Study
Source: PLoS One. 2008 Oct 9;3(10):e3363. doi: 10.1371/journal.pone.0003363 (PMC2566605; doi:10.1371/journal.pone.0003363)
Supplement: Amendment S1 — Amendment september 2008 to the protocol and statistical analysis plan. (0.06 MB DOC) [file pone.0003363.s004.doc]

15.09.2008.

Amendment to the statistical analysis plan and study protocol file for the study entitled:

**“Effect of Metformin on glycaemic control and non-glycaemic cardiovascular risk factors in patients with type-1 diabetes and persistent unacceptable regulation on treatment with insulin and diet.”**

**The following errors have been corrected in the database:**

1) Patient no. 10008, 10031 and 10044:

Laboratory data on secondary outcomes (e.g. lipid-levels, body weight and safety data) measured at the drop-out date (or earlier) for last-observation carry forward have been included. These data were obtained in the non-fasting state from non-study visits in the outpatient clinic.

2) Patient no. 10031 and 10079:

The insulin dose at the drop-out date was stated inconsistently in two separate tables in the database and has therefore been corrected in accordance with the case-report form/patient records. For patient no. 10031 the morning insulin dose at the date of the last telephone consultation (03.03.2005) has been corrected from 28 units to 36 units. For patient no. 10079 the basal insulin dose at the drop-out date (19.01.2006) has been corrected from 24 to 24,3 units (continuous insulin infusions).

3) Patient no. 10008 and 10055:

In the placebo group, these two patients experienced diabetic ketoacidosis (DKA) probably precipitated by myocardial infarction (10008) and gastroenteritis (10055), respectively. In the database and the case-report form, these two cases of DKA together with their corresponding precipitating events of myocardial infarction and gastroenteritis, respectively, were registered as two single serious adverse events (SAE). Due to myocardial infarction and gastroenteritis being the precipitating events of DKA, in the database and the case-report form, these two SAE’s were both registered as not being potentially related to the study drugs (prior to unmasking). However, in order to reflect the potentially relation to the study-drugs for the two cases of DKA (prior to unmasking), in the database and the case-report form, there have been made separate SAE registrations for each of these. Hereafter the events of myocardial infarction and gastroenteritis remain registered as two separate SAE’s, each without being potentially related to the study drugs, whereas the two events of DKA are registered as two separate SAE’s, each with potential relation to the study drugs (prior to unmasking). Hence, for each of patient no. 10008 and 10055, one potentially study drug related SAE (DKA) has been added.

4) Patient no. 10050:

In accordance with the case report form, this patients’ estimation of allocated treatment (at the end of treatment) have been corrected from “missing” to “metformin”.

**Implications for the statistical analyses:**

The additional secondary outcome laboratory data as mentioned in 1) to be analyzed for patient 10008, 10031 and 10044 have been censored to include only safety parameters (e.g. blood concentrations of albumin, haemoglobin, creatinine, sodium, potassium, bicarbonate, cobalamin, folate, alkaline phosphatase, aspartate aminotransferase, factor II VII X, platelet and white blood cell counts as well as urinary ketone-bodies). Since these data were obtained in the non-fasting state (and not in the fasting state as data from other study visits), the secondary outcome data (e.g. plasma glucose, lipid levels, body weight, blood pressure etc.), besides safety data (and adverse events – see 10) below), from these patient visits will not be included in the statistical analysis. Levels of haemoglobin A1c (the primary outcome) from these patient visits have been included in the database previously and are therefore not affected by these changes.

**In the process of updating the database the following additional information have been made available in accordance with the case-report form/patient records:**

5) The database did not systematically reflect patients who made changes in the insulin regimen by changing insulin preparations as well as the number or site of insulin injections (only one of each of these categories was available for each patient). The information about these changes were available in the database as individual comments and these have therefore been systematically re-coded to also reflect such changes in insulin preparation and the number/site of injections.

6) For all patients:

Data on doses (including dates of changes) and generic/trade names of concomitant medications (i.e. antihypertensive medications, potassium supplementations, aspirin, non-steroid anti-inflammatory drugs and lipid-lowering therapy) have been included in the database. Also, diuretics have been divided into separate categories of thiazide and loop diurects as well as aldosterone antagonists.

7) Patient no. 10002, 10005, 10022, 10026, 10029, 10032, 10051, 10057, 10060, 10062, 10088 and 10101:

Data on starting and/or stopping of concomitant medications (i.e. ACE-inhibitors (patient no. 10005, 100051 and 10057), angiotensin II receptor blockers (patient no. 10060 and 10101), beta-blockers (patient no. 10029), calcium antagonists (patient no. 10022, 10026 and 10051), diuretics (patient no. 10032, 10088 and 10101), potassium supplementations (patient no. 10002, 10062, 10088 and 10101), aspirin (patient no. 10088), systemic steroid treatment (patient no. 10026) and other medications (patient no. 10026 and 10088) have been corrected.

8) For all patients:

Data on insulin doses not taken permanently (i.e. additional daily insulin doses termed as “pn” doses) have been corrected for the drop-out patients (i.e. patient no. 10031, 10054, 10064, 10068, 10071, 10078, 10079, 10097 and 10111), but not for other patients. These corrections have not been done for all patients since these additional non-permanent insulin doses are not included in the calculation of the total daily insulin dose and have therefore no influence on the conclusions from the study.

9) For all SAE’s, separate categories have been made to reflect the expected primary cause of the event – for example, cardiovascular, neurological, infections etc.

10) For all drop-out patients, adverse event data have been made available from the date of the last contact in the study and until the drop-out date.

11) For individual patients, the following changes have been made:

Patient no. 10002: The stopping date of gastrointestinal symptoms (flatulence) has been corrected from 28-10-2005 to 26-10-2005.

Patient no. 10008: The level of potassium (7,7 mmol/l) at admission for myocardial infarction and DKA (during placebo treatment) have been added to the comments.

Patient no. 10037: The onset date of gastrointestinal symptoms (flatulence) has been corrected from 07-12-2005 to 07-12-2004.

Patient no. 10038 and 10106: The date of the last dose of the study-medication has been corrected from 04-01-2005 to 04-01-2006 for patient no. 10038 and from 01-06-2005 to 01-06-2006 for patient no. 10106.

Patient no. 10048: Nausea was registered as an adverse event during the run-in period as well as during the randomized period. Only the latter is correct and the occurrence has therefore been deleted from the run-in period.

Patient no. 10059: Symptoms of blurred vision and vasomotor symptoms (hot flashes) have been added to the adverse events comments.

Patient no. 10063: An event of syncope, of unknown cause, has been changed from a cardiovascular to a neurological adverse event (in accordance with events of syncope in other patients).

Patient no. 10072: The stop-date (16-03-2005) of the adverse event of itching was earlier than the onset-date (01-05-2005). The stop-date could not be unequivocally identified from the case-report form/patient records and has therefore been deleted.

Patient no. 10097: Gastrointestinal bleeding has been amended as the cause of death (during the run-in period).

12) The secondary outcomes of fasting plasma glucose and bicarbonate were not pre-specified in the original study-protocol or amendments hitherto. However, both of these variables were, from the first-patient first visit-date, measured at identical schedules as for other (pre-specified) outcomes (please, see the copy of the blood-sampling schedule for each study-visit at the end of this amendment – English translation). Due to these conditions, we therefore consider fasting plasma glucose and bicarbonate as pre-specified variables.

Søren Søgaard Lund, M.D.

Peter Rossing, DMSc, Chief Physician.

Allan Arthur Vaag, PhD, DMSc, Chief Physician, Professor.

Steno Diabetes Center, Gentofte, Denmark.

**Met-1**

GCP-unit stamp

Pt no |___||___||___||___||___|

Pt init |___||___||___|

*Label*

Visit no. 02

Study-visit date |___||___|/|___||___|/|___||___|

dd mm year

| **Height:** |___||___||___| cm  **Body-Weight:**|___||___||___|,|___| kg  ( wearing only underwear ) | P-glucose: |___||___|,|___| mmol/l  (measured by Precision Xtra) | **Hip-Waist-ratio:**  Waist: |___||___||___| cm  Hip: |___||___||___| cm |
| --- | --- | --- |

| **BP** sitting position, automatic BP-device. | | **pulse** |
| --- | --- | --- |
| Right Arm (mmHg) | |___||___||___| / |___||___||___| | |___||___||___| |
| Left Arm (mmHg) | |___||___||___| / |___||___||___| | |___||___||___| |

| **Bloodsample 1 (520-37)**  Label 1  **DNA**  **Extra serum**  **Extra plasma**  **2 x citrateplasma**  **2 x EDTA plasma**  **2 x serum**  **Methylglycoxylate**  **t-PA-antigen**  **PAI-activity**  **PAI-antigen**  **t-PA-activity**  **Glucose**  **HbA1C**  **Multistix7**  **Body Weight**  **Height**  **Keto**  **Haemoglobin**  **B-White Blood Cell Count**  **B-Platelet Count**  **MPV**  **Bicarbonate**  **Potassium**  **Sodium**  **Creatinine**  **Albumin**  **ASAT**  **Lipids**  **Factor 2,7,10**  **Cobalamin**  **Ery-Folate**  **Alkaline phosphatase** | **Bloodsample 2 (520-37)**  Label 2  **HbA1C** |
| --- | --- |
|  | **24hour urine x 3 (520-37)**  Urinelabel 1  Urinelabel 2  Urinelabel 3  **U-Albx**  **24hU-Alb**  **24hU-So,Crea,Carb(Urine 1)**  **2xStorageurine (4,5 ml)**  ** Received**  ** Send** |

CRF version #1

Page 9 of 34
